# Value of Glucosylsphingosine (Lyso-Gb1) as a Biomarker in Gaucher Disease: A Systematic Literature Review

**DOI:** 10.3390/ijms21197159

**Published:** 2020-09-28

**Authors:** Shoshana Revel-Vilk, Maria Fuller, Ari Zimran

**Affiliations:** 1 Gaucher Unit, Shaare Zedek Medical Center, Shmu’el Bait St 12, Jerusalem 9103102, Israel; srevelvilk@gmail.com (S.R.-V.); azimran@gmail.com (A.Z.); 2 School of Medicine, Hebrew University, Jerusalem 9112102, Israel; 3 Genetics and Molecular Pathology, SA Pathology at Women’s and Children’s Hospital, North Adelaide, SA 5006, Australia; 4 School of Medicine, University of Adelaide, Adelaide, SA 5005, Australia

**Keywords:** Gaucher disease, lyso-Gb1, glucosylsphingosine, biomarker, systematic literature review, lysosomal storage disorder

## Abstract

The challenges in the diagnosis, prognosis, and monitoring of Gaucher disease (GD), an autosomal recessive inborn error of glycosphingolipid metabolism, can negatively impact clinical outcomes. This systematic literature review evaluated the value of glucosylsphingosine (lyso-Gb1), as the most reliable biomarker currently available for the diagnosis, prognosis, and disease/treatment monitoring of patients with GD. Literature searches were conducted using MEDLINE, Embase, PubMed, ScienceOpen, Science.gov, Biological Abstracts, and Sci-Hub to identify original research articles relevant to lyso-Gb1 and GD published before March 2019. Seventy-four articles met the inclusion criteria, encompassing 56 related to pathology and 21 related to clinical biomarkers. Evidence for lyso-Gb1 as a pathogenic mediator of GD was unequivocal, although its precise role requires further elucidation. Lyso-Gb1 was deemed a statistically reliable diagnostic and pharmacodynamic biomarker in GD. Evidence supports lyso-Gb1 as a disease-monitoring biomarker for GD, and some evidence supports lyso-Gb1 as a prognostic biomarker, but further study is required. Lyso-Gb1 meets the criteria for a biomarker as it is easily accessible and reliably quantifiable in plasma and dried blood spots, enables the elucidation of GD molecular pathogenesis, is diagnostically valuable, and reflects therapeutic responses. Evidentiary standards appropriate for verifying inter-laboratory lyso-Gb1 concentrations in plasma and in other anatomical sites are needed.

## 1. Introduction

Gaucher disease (GD) is an autosomal recessive disease of glycosphingolipid metabolism caused by a functional deficiency of the lysosomal enzyme β-glucocerebrosidase (glucosylceramidase (GBA); EC 3.2.1.45), resulting from variants in the *GBA1* gene [1,2]. GD is one of the more common lysosomal storage disorders, occurring with an incidence of approximately 1 in 50,000 to 100,000 live births [3,4], although genetic studies indicate a homozygote frequency of ∼1:850 in the Ashkenazi Jewish population [5].

GBA deficiency results in the progressive accumulation of the substrate glucosylceramide (Gb1), along with a build-up of related glycosphingolipids almost exclusively in cells of the mononuclear phagocyte (reticulo-endothelial) system [6,7]. These transform into Gaucher cells, which have a distinct macrophage phenotype with a characteristic morphology and are metabolically active and alternatively activated [8]. Although GD presents as a continuum of phenotypes, the disease is typically categorized into three main types. Type 1 GD (OMIM #230800), the non-neuronopathic and most prevalent variant among Caucasians, is a multisystem inflammatory disorder most often characterized by major liver, spleen, bone, and hematological pathology [1,9]. Lung involvement, pulmonary hypertension, and renal involvement are rarer presentations [1]. Type 2 GD (OMIM #230900) is the acute neuronopathic variant, occurring in very young children, and type 3 GD (OMIM #231000) is a subacute neuronopathic variant that tends to manifest neurologically in childhood or adolescence [1].

One of the challenges in the care of patients with GD is the development of biomarkers as validated tools that aid diagnosis, prognosis, follow-up, treatment decisions, and pathophysiologic understanding [6,10,11]. A biomarker is defined by the National Institutes of Health (NIH) Biomarkers Definitions Working Group as “a characteristic that is objectively measured and evaluated as an indicator of normal biological processes, pathogenic processes, or pharmacologic responses to a therapeutic intervention” [12]. In the era of newborn screening for GD, the use of a reliable biomarker in combination with testing for GBA activity has the potential to reduce the false positive rate and inform decisions about when to start therapy. Patients with GD require continual monitoring and follow-up. Ideally, a real-time pharmacodynamic biomarker with the capacity to identify non-responses could effect changes in management (e.g., dose adaptation) promptly, such that disease progression and organ damage could be avoided.

Selecting appropriate candidate biomarkers has been hampered by the complexity of this monogenetic disorder, in which modifier genes, epigenetics, and external factors give rise to vast clinical and biochemical heterogeneity [6,13,14,15,16,17]. This phenomenon partly explains why there are such poor correlations between the *GBA* genotype and residual GBA activity as a diagnostic and prognostic tool [6]. Historical plasma biomarkers for disease monitoring and evaluation of treatment (i.e., tartrate-resistant acid phosphatase, angiotensin-converting enzyme, ferritin, and alkaline phosphatase) are not specific to GD, are only elevated moderately in patients with GD relative to controls and are influenced by other factors [18,19,20,21,22,23].

The utility of more widely used plasma biomarkers is also limited. The hydrolase chitotriosidase and the chemokine (C-C motif) ligand 18 (CCL18) are secreted by activated macrophages, including Gaucher cells [23,24], and are thus indicative of overall Gaucher cell burden. Although chitotriosidase and CCL18 are elevated in patients with GD relative to healthy controls and decrease during disease-specific treatment [22,23,24,25,26,27,28], neither biomarker is central to disease pathophysiology [24]. Furthermore, chitotriosidase and CCL18 are not specific for GD [24,29] and one in 20 individuals are entirely deficient in chitotriosidase activity owing to homozygosity for the 24-base-pair duplication in the *CHIT1* gene [30].

Over the last half-century, there has been ongoing focus on the occurrence of the sphingoid base in the primary lysosomal storage lipids as mediators of disease [6,11,31]. Gaucher cells are laden with lipids and are known to secrete an array of macromolecules into the local environment they infiltrate [32]. The presence of Gaucher cells along with exposure of tissue to their secretome is thought to partly explain the multifaceted characteristics of the disease [32]. In GD, there is evidence that elevated levels of the lysosomal storage product and sphingolipid, glucosylsphingosine (lyso-Gb1), a direct metabolite of Gb1, are pathogenic to cells [6,7,33].

### 1.1. Gb1 Metabolism and Lyso-Gb1

**Figure 1:**
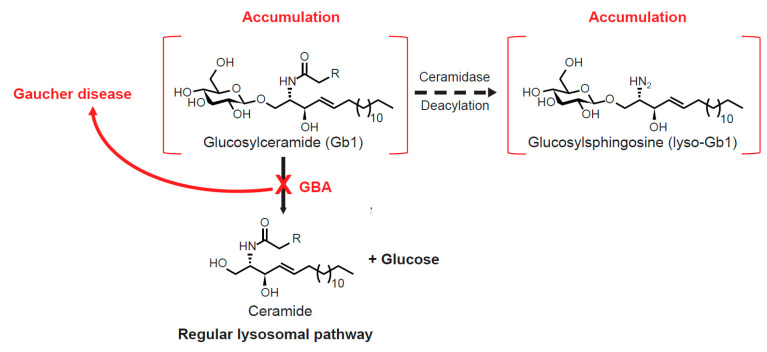
Catabolic route of glycosphingolipid generation in Gaucher disease [7].

There are at least three adaptations in Gb1 metabolism that occur during GBA deficiency—namely, the increased anabolism of Gb1 to gangliosides, excessive transglycosylation by cytosol-faced retaining β-glucosidase GBA2, and the active deacylation of Gb1 by acid ceramidase to lyso-Gb1 (the latter pathway is illustrated in Figure 1) [7]. The differential activation of these pathways may account, at least in part, for the inconsistencies observed in the translation of the *GBA* genotype, lipid storage deposition, and GD phenotype [6]. Variable alterations in lipid metabolism secondary to Gb1 (and lyso-Gb1) accumulation may also induce multiple pathologies reflected in phenotypic heterogeneity [34].

**Figure 2:**
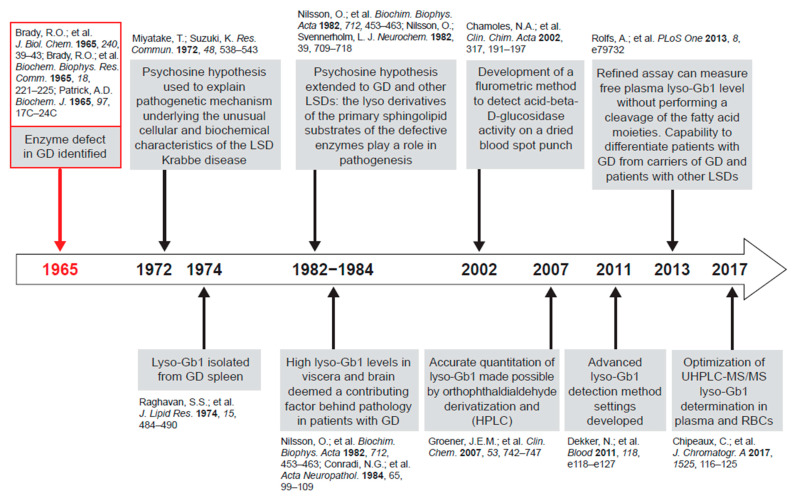
Research milestones culminating in glucosylsphingosine (lyso-Gb1) as a focus of biomarker research.

Lyso-Gb1, being more hydrophilic than Gb1, has physicochemical characteristics likely permitting egress from the lysosomal system into the cytoplasm and extracellular space [6]. Results of studies across different research settings over the last 50 years implicate the acid ceramidase pathway in GD because supraphysiological lyso-Gb1 concentrations are present at specific anatomical sites in affected patients and in animal models of GD (Figure 2) [6,7,11]. Furthermore, elevated lyso-Gb1 is associated with the development of GD pathology [6].

A close examination of lyso-Gb1 as a pathogenic metabolite in GD was made possible in 2007 when the quantitation of lyso-Gb1 in biological samples was significantly improved by orthophthaldialdehyde derivatization and high-performance liquid chromatography [35]. Further improvement to the technique was achieved by the development of a liquid chromatography tandem mass spectrometry (LC/MS/MS) method using an identical radiolabeled Gb1 standard [7]. The validation and clinical utility of this method was demonstrated in a prospective observational study of a cohort of patients with type 1 GD and controls [33].

### 1.2. Systematic Literature Review Objectives

In light of recent research efforts in this area, we undertook what we believe is the first systematic review of the published literature to report on lyso-Gb1 as a potential biomarker for diagnosis, prognosis, and disease/treatment monitoring. The review scrutinizes the role of lyso-Gb1 in GD pathophysiology and as a bona fide biomarker that has a central role in this process.

## 2. Results

### 2.1. Search Results

**Figure 3:**
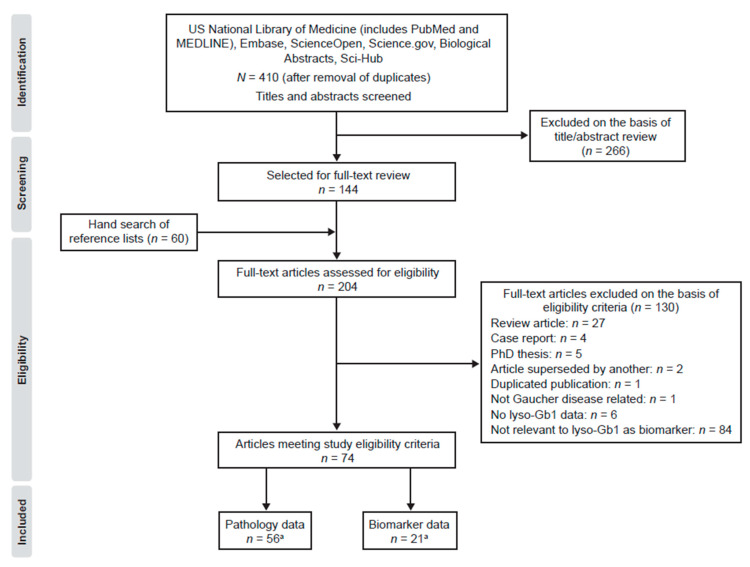
Literature identification and study selection process for publications reporting on glucosylsphingosine (lyso-Gb1) in Gaucher disease (PRISMA flowchart). ^a^: Three articles reported on roles for lyso-Gb1 in pathophysiology and as a biomarker [26,45,53].

The literature searches on lyso-Gb1 and GD identified 410 articles (Figure 3). During title and abstract screening, 266 articles were excluded according to study exclusion criteria. One hundred and forty-four articles were retrieved in full text, and an additional 60 articles meeting inclusion criteria were identified from hand searches of reference lists from full text articles. Of the 204 articles that were reviewed in full text, 130 were excluded, primarily owing to article type or for their lack of relevance to lyso-Gb1 as a biomarker for GD. Thus, 74 articles met the study eligibility criteria and were included in the review. Of these, one was a randomized clinical trial, one was a pooled analysis of phase III trials, 29 were non-randomized clinical trials or observational studies, and 48 were preclinical studies (five publications reported both preclinical and clinical data).

### 2.2. Quality Assessment

Using the NICE STA method, risk of bias in the sole randomized controlled trial was deemed low, although bias regarding the allocation concealment process and between-group baseline similarities was unclear (Table S1) [36]. Thirty non-randomized clinical trials and observational studies with full-study reporting were assessed using the Newcastle–Ottawa scoring tool [3,26,33,37,38,39,40,41,42,43,44,45,46,47,48,49,50,51,52,53,54,55,56,57,58,59,60,61,62,63], of which 19 were deemed to have a high risk of bias (score 0–3; Table S2). Thirty-two animal studies were assessed using the SYRCLE risk of bias tool [45,53,58,64,65,66,67,68,69,70,71,72,73,74,75,76,77,78,79,80,81,82,83,84,85,86,87,88,89,90,91,92]; 20 were considered low-risk, nine high-risk, and the level of bias was unclear in the remaining three studies (Table S3). Other preclinical studies could not be quality assessed owing to a lack of suitable validated tools.

### 2.3. Pathology

Of the 74 articles included, 56 articles pertained to lyso-Gb1 and GD pathology. They encompassed (in a non-mutually exclusive manner) ten clinical (autopsy) studies [54,55,56,57,58,59,61,62,63,93] and 48 studies reporting preclinical data (19 studies reporting in vitro data and 32 studies reporting in vivo data) [26,45,53,58,63,64,65,66,67,68,69,70,71,72,73,74,75,76,77,78,79,80,81,82,83,84,85,86,87,88,89,90,91,92,94,95,96,97,98,99,100,101,102,103,104,105,106,107].

#### 2.3.1. Accumulation of Lyso-Gb1 in Gaucher Disease

Evidence for lyso-Gb1 accumulation consistent with the pathology of GD was found in all ten autopsy studies (Table 1) [54,55,56,57,58,59,61,62,63,93]. Large quantities of lyso-Gb1 were found in the spleen and liver of deceased patients with GD [54,55,93] and lyso-Gb1 had accumulated in the cerebrum and cerebellar cortices of patients with types 2 and 3 GD with severe neurodegeneration [56,57,58,59,61,62,63]. Elevated lyso-Gb1 was not present in one patient with non-neuronopathic GD [57,61]; however, brain lyso-Gb1 was found in large amounts in the cerebellar cortex of severely affected patients with type 1 GD [54]. Lyso-Gb1 was detected in the brain, liver, and spleen tissue of human fetuses with type 2 GD [58].

Seven of eight in vitro studies provided evidence for a role of lyso-Gb1 accumulation in GD (Table S4). In cultured human cells collected from patients with types 1 and 2 GD, intracellular lyso-Gb1 was substantially higher than in cells from healthy control subjects [26,94,103,108]. These observations were recapitulated when control cells were exposed to conduritol B epoxide (CBE) [26,99,103], a specific GBA inhibitor, and reversed when Gaucher cells were exposed to non-inhibitory chaperones of GBA or acid ceramidase inhibition and CBE removal [26,94,108]. Taken together, these findings confirm the intralysosomal conversion of accumulating Gb1 to lyso-Gb1 [26]. A human in vitro study that reported normal lyso-Gb1 concentrations and lyso-Gb1 hydrolysis in human fibroblasts isolated from patients with types 1, 2, and 3 GD was rationalized by a relatively high residual activity of GBA versus that found in other cells [99]. Intracellular lyso-Gb1 also accumulated in *GBA* knockout mice models and in newborn neural cells from a type 3 GD mouse model in the presence of CBE [84,101,102].

Table S5 shows that all 31 in vivo studies of experimental GD provided evidence of lyso-Gb1 accumulation, with eight studies citing a reduction in lyso-Gb1 after exposure to GBA or promoters of GBA expression [66,89], or a removal of factors promoting the inhibition of GBA expression [76,79,81,82,90,92].

In all five pharmacologically induced GD murine models, lyso-Gb1 was significantly elevated in the brain after exposure to the GBA inhibitor CBE [64,72,76,81,85], and in the liver and spleen of one model with cyclophellitol [64]. However, these agents may have effects on additional targets: both cyclophellitol and CBE have been shown to inhibit GBA2 as well as GBA, although CBE only at significantly higher concentrations than those used for GBA [109,110].

In type 1 GD murine models, lyso-Gb1 was elevated in the spleen and liver [45,66,71,77,80,92], blood [79,80], and bone [66], but not brain. Lyso-Gb1 declined after mice were exposed to eliglustat, a potent and selective inhibitor of glucosylceramide synthase [79]. Lyso-Gb1 was also significantly decreased in the bone marrow, spleen, and liver of mice with an induced deletion in the Gba genes (Mx1-Cre^+^ Gba^flox/flox^) treated with therapeutic vectors containing the Gba gene, relative to control mice [66].

Lyso-Gb1 accumulation was observed in the brain [53,65,67,68,69,70,72,73,76,82,83,84,85,86,88,89,90,91,111], viscera [65,73,86,91], and blood [111], but not bone, and in a wide range of neuronopathic GD murine models and one ovine model. Although murine models are highly economical, lamb brain is considered more translationally relevant to the human brain as both are gyrencephalic and have similar anatomy and physiological vital signs. In a newborn lamb model of type 2 GD harboring a pathogenic C381Y mutation that corresponds to the same mutation in humans with a similar clinical outcome, lyso-Gb1 concentrations were several orders of magnitude higher in the brain, cervical spinal cord, spleen, and liver than in corresponding samples from wild-type and heterozygous lambs [73].

#### 2.3.2. Relationship between Lyso-Gb1 and GD Pathology

GD pathology is marked by the presence of Gaucher cells, neuropathology, and chronic low-grade inflammation [112]. Evidence for lyso-Gb1 as a pathogenic mediator in GD based on an association with these features was found in all 16 in vitro studies (Table 2) and 14 of 17 in vivo studies (Table 3). With the exception of one *in vivo* study [45], the effects of lyso-Gb1 on cells and tissues were always damaging, ranging from moderate to high cytotoxicity depending on lyso-Gb1 concentration and the type of cell or tissue under study. It is important to bear in mind that in preclinical studies examining the effects of exogenous lyso-Gb1, concentrations of lyso-Gb1 used were lower than the elevated intracellular lyso-Gb1 seen in some animal models and patients with GD.

From a mechanistic viewpoint at the cellular level, lyso-Gb1 evoked HEK293 and RH7777 cells to form globoid cells (multinucleated macrophages) [106], a common manifestation of GD, via the activation of the G protein–coupled receptor T cell-associated gene 8 (TDAG8). Binding of TDAG8 by lyso-Gb1 promoted inflammation via the activation of phospholipase A2 [104]. Immortalized *GBA*^−/−^ neurons had marked accumulation of Gb1 and lyso-Gb1, enlarged lysosomes, and an impaired ATP-dependent calcium-influx response [101]. A wide range of in vitro experiments has also demonstrated that lyso-Gb1 is cytotoxic to some cell types and pro-inflammatory in others, is damaging to specific neurons, impairs cell fission during cytokinesis, and interferes with osteoblasts, immune regulation, and signal transduction (Table 2). Exogenous lyso-Gb1 induced hemolysis and was cytotoxic towards cultured human cholinergic neurons, human umbilical vein endothelial cells, and 3T3 and PC12 cell lines [64,83,100,107]. Lyso-Gb1 toxicity towards fibroblasts and neural crest-derived neoplastic cells in vitro occurred at a similar concentration to that which it accumulates in vivo [64].

Over the past two decades, it has become apparent that there is an association between GD and Parkinson disease, with both diseases sharing neuropathological features [10]. Lyso-Gb1 promoted not only the elevation of α-synuclein levels in neurons from patients with type 2 GD or Parkinsonism but also the formation of toxic oligomeric α-synuclein aggregates [84,102,108], a hallmark of Parkinson disease [10].

Results from 14 of 17 experimental animal models of GD have shown that lyso-Gb1 is associated with: pro-inflammatory effects [75]; hepatosplenomegaly [67,74,75,77]; poor hematologic [75,77], skeletal [77], and neurologic outcomes [68,76,82,83]; B-cell malignancy [79]; death [89,90] (Table 3). Two in vivo studies did not evaluate the effect of lyso-Gb1 in specific aspects of GD pathology [45,78].

In the CBE-induced murine GD model, high levels of lyso-Gb1 alone or in combination with Gb1 were accompanied by brain α-synuclein aggregation, neurodegeneration, microglia and complement C1q activation, and premature death [81,85], with gliosis and neurobehavioral deficits reversed by the specific glucosylceramide synthase inhibitor venglustat (ibiglustat) [76].

In a lyso-Gb1-induced GD model reflecting lyso-Gb1 levels observed in moderate to severely affected patients with untreated GD, decreased hemoglobin (Hb) and hematocrit was observed along with hepatosplenomegaly [75]. In addition, there was a strong increase in CD68 immunoreactivity in the spleen sections of lyso-Gb1-treated mice when compared with vehicle controls [75].

Most pathologic associations of lyso-Gb1 in the conditional type 1 GD model were incidental, although two studies using the *GBA1* knockout mouse model (Mx1-Cre^+^) detected a correlation between splenic lyso-Gb1 levels and splenomegaly [74,77]. Interestingly, this clinical phenotype, including the bone pathology, was rescued by *GBA2* knockout despite elevated Gb1 and lyso-Gb1 levels [45]. These data implicate lyso-Gb1 as a source of sphingosine generation in aspects of GD pathology.

Similar to the type 1 GD models, most associations of lyso-Gb1 with pathology in the neuronopathic variants were incidental (Table 3). In the two correlational analyses, there was no association detected between brain concentrations of lyso-Gb1 and neuronal loss in the neuronopathic GD murine *GBA^flox/flox^*; nestin-Cre model [70], whereas an intracerebroventricular administration of recombinant human GBA resulted in dose-dependent reductions in lyso-Gb1 in the brain samples of neonatal K-14Cre^+^ *GBA^lnl/lnl^* mice and improved survival [89].

Preclinical data and evidence from 20 patients with GD showed that the frequency of lyso-Gb1-specific T cells correlates with disease activity and therapeutic response [113].

Excessive lyso-Gb1 has a role in dysregulating humoral immunity by promoting chronic B-cell activation and gammopathy, which can evolve into multiple myeloma, a relatively common blood cancer in patients with GD [113].

### 2.4. Lyso-Gb1 as a Clinical Biomarker

Of the 74 articles included in the analysis, 21 reported data that pertained to plasma lyso-Gb1 as a biomarker in GD.

#### 2.4.1. Diagnosis

Eleven studies provided information relevant to the diagnosis of GD [3,26,33,38,40,41,44,46,47,49,50]. Normally, lyso-Gb1 is undetectable or found at trace levels in plasma (i.e., <4.9 ng/mL (10.61 nmol/L)) and tissue [39,114]. Data from five prospective observational studies indicated consistently that lyso-Gb1 in plasma and red blood cell (RBC) membranes were higher in untreated patients with GD than in control subjects (Table 4) [3,26,40,44,46]. In addition, findings from an open-label pilot clinical trial showed that lyso-Gb1 in cerebrospinal fluid were below the lower limit of quantification (10.0 pg/mL (21.66 pmol/mL)) in all control subjects but were elevated in patients with neuronopathic GD [49]. An additional five observational studies were specifically designed to assess the value of lyso-Gb1 as a diagnostic biomarker (Table 4) [33,38,41,47,50]. All utilized sensitive LC/MS/MS techniques for the detection and quantification of lyso-Gb1 in plasma.

Two groups, Dekker et al. [33] and Rolfs et al. [50] reported on the use of lyso-Gb1 as a biomarker for patients with GD. Dekker et al. prospectively recruited 64 patients with GD, 34 obligate carriers of GD, and 28 healthy controls from centers in the Netherlands, United States, and Poland. Most of the patients with GD were compound heterozygotes for N370S (c.1226A > G) and one other *GBA* mutation, with the exception of five patients homozygous for N370S. Fourteen homozygotes for the N370S mutation were also studied, two patients with saposin C deficiency, and three patients with type 3 GD [33]. Prominent increases in lyso-Gb1 (300-fold) were detected in the plasma of symptomatic patients with type 1 GD versus healthy controls. The extent of this abnormality was emphasized by the plasma Gb1 level, which was only three-fold higher in these patients with type 1 GD [33].

Rolfs et al. retrospectively analyzed non-Jewish Caucasian patients from a single center in Germany [50]. The marker was specific for GD, as carriers and patients with other lysosomal storage disorders did not show signs of elevated lyso-Gb1. A plasma lyso-Gb1 threshold of 12 ng/mL (25.99 nmol/mL) differentiated between patients with genetically defined GD from healthy probands, patients with other lysosomal storage diseases, and GD carriers, with 100% sensitivity and 100% specificity [50]. The observation was also independent of sex, as male and female patients with GD had similar lyso-Gb1 levels [50].

In a US study, Murugesan et al. prospectively compared plasma lyso-Gb1 in patients with type 1 GD and healthy controls. In this cohort, the investigators found that a lyso-Gb1 cut-off of 4 ng/mL had 100% sensitivity and specificity as a diagnostic tool [47]. The different diagnostic cut-off values for plasma lyso-Gb1 thus reflect the different populations under study and different means of lyso-Gb1 measurement.

Fuller et al. described an accurate, reproducible, robust, and easy-to-perform assay for the determination of plasma lyso-Gb1 concentrations in a routine laboratory setting using N-palmitoyl-d3-lactosyl ceramide as an internal standard [41]. This assay reported a performance of 100% sensitivity and specificity using a lyso-Gb1 cut-off of 4 pmol/mL (1.85 ng/mL).

Chipeaux et al. prospectively recruited 64 patients with GD, 34 obligate carriers of GD, and 28 healthy controls from three centers in France [38]. Plasma lyso-Gb1 (limit of quantification, 0.7 nM) was the only relevant biomarker in both plasma and RBCs for GD diagnosis when compared with Gb1, sphingosine, and sphingosine-1-phosphate [38].

The findings of one additional study showed that it is possible for patients other than those with GD to have high lyso-Gb1 [42]. In patients with action myoclonus-renal failure (AMRF), deficiency of the lysosomal integral membrane protein-2 prevents the cell type-specific trafficking of GBA to lysosomes. GBA is almost absent in the lysosomes of AMRF fibroblasts, but is present in white blood cells. As a result, AMRF macrophages can still produce lyso-Gb1, but patients with AMRF do not present with Gaucher cells, and do not have elevated macrophage markers such as chitotriosidase [42], important factors for consideration in diagnostic assay specificity.

#### 2.4.2. Prognosis

Four studies provided information on lyso-Gb1 as a prognostic biomarker of GD (Table 5) [26,33,39,50].

In a prospective, case-control study, modest increases in plasma lyso-Gb1 were evident in mildly affected patients [33]. Plasma lyso-Gb1 concentration was correlated with the genotype (N370S *GBA* homozygous patients only), liver volume, and bone marrow fat fraction but not correlated with macrophage inflammatory protein 1-β, the presence of a spleen, skeletal complications, osteocalcin, and procollagen type 1 N propeptide in this study [33].

In a retrospective, case-control study, plasma lyso-Gb1 appeared to reflect the severity of the individual genetic variant [50]. Patients with N370S displayed lower concentrations of lyso-Gb1 than those with L444P (c.1448T > C), which is known to be more frequent in patients with a more fatal course of disease [50,115]. An exploratory pooled analysis of phase III clinical trials revealed that mean plasma lyso-Gb1 was twice as high for 17 patients with ≥1 allele with the N370S variant (N370S/N370S or N370S/other) than for five patients with non-N370S mutations [39].

Lyso-Gb1 concentrations were also excessive in the cultured fibroblasts of a collodion patient with GD (homozygous for the recombination RecNci allele) with virtually no residual GBA activity versus more modest elevations among patients with type 1 GD and neuronopathic variants [26].

#### 2.4.3. Disease Monitoring/Responsivity to Treatment

In 17 studies, cross-sectional and longitudinal data on lyso-Gb1 in treated and untreated patients with GD were compared [33,36,37,38,39,40,41,43,44,45,47,48,49,50,51,52,53]. Results from 16 of the 17 studies showed that treatment with enzyme replacement therapy (ERT) and substrate reduction therapy (SRT) either alone or in combination produced marked reductions in lyso-Gb1 concentrations in plasma, cerebrospinal fluid (CSF), and urine relative to baseline or control (Table S6). For most patients, a significant reduction in plasma lyso-Gb1 occurred after ERT initiation before levelling off at a lower concentration and increasing when ERT was stopped [39,50]. Similar findings were observed after the initiation of SRT [43]. A retrospective analysis of 25 non-splenectomized patients with GD homozygous for the non-neuronopathic N370S GBA mutation in the *GBA1* gene who received low-dose ERT (15 units/kg/month) revealed an exponential decay (Pearson product moment determination coefficient (*r*^2^), 0.84) in lyso-Gb1 plasma over 72 months [37]. ERT also lowered lyso-Gb1 content in RBC membranes in a prospective, multicenter, cross-sectional, case-control study of 15 patients with type 1 and neuronopathic GD receiving ERT versus 16 untreated counterparts [40]. A combination of ERT plus ambroxol reduced lyso-Gb1 concentration in CSF by 26% versus baseline in a multicenter open-label pilot study of five patients with neuronopathic GD; concentrations were below the lower limit of quantification (10.0 pg/mL) in all 37 control subjects [49]. However, in all studies, the lyso-Gb1 concentration remained higher than that observed in healthy controls regardless of sample type, and higher than the 4 or 12 ng/mL diagnostic cut-off level for lyso-Gb1 in plasma (Table S6).

Ten of the 17 studies provided different evidence levels of information on lyso-Gb1 as a response biomarker in GD (Table 6) [36,37,38,39,40,43,44,47,49,51]. The results of a prospective, multicenter, cross-sectional, case-control study in treatment-naïve and splenectomized patients with type 1 GD showed that lyso-Gb1 concentrations in plasma (Pearson product moment correlation coefficient (*r*), –0.83) and RBC membranes (−0.65) correlated inversely with hematocrit [38]. Nine studies were of patients with GD undergoing treatment with ERT, SRT, or ambroxol [36,37,39,40,43,44,47,49,51]. Five of the associations between lyso-Gb1 and clinical outcomes were incidental, albeit with strong temporal and kinetic links, whereas the other five studies demonstrated correlations between lyso-Gb1 and clinical outcomes.

Regarding incidental findings in the periphery, as plasma lyso-Gb1 decreased upon treatment with either ERT or SRT, platelet counts, Hb, and the bone marrow fat fraction increased, whereas spleen and liver volumes decreased [36,37,43,51]. The lumbar spine T-score also increased to the normal range from baseline as plasma lyso-Gb1 decreased owing to SRT in a phase II multicenter clinical trial [43]. Centrally, as CSF lyso-Gb1 concentrations decreased among five patients with neuronopathic GD receiving ERT plus ambroxol, myoclonus, seizures, and pupillary light reflex dysfunction markedly improved [49].

With respect to correlations, an exploratory pooled analysis of phase III clinical trials of treatment-naïve patients with type 1 GD receiving ERT revealed that there was moderate pairing between decreasing plasma lyso-Gb1 concentrations and increasing platelet counts at weeks 13 (*r*, −0.530), 25 (*r*, −0.654), and 53 (*r*, −0.503), and between decreasing plasma lyso-Gb1 concentrations and decreasing spleen volumes at weeks 25 (*r*, 0.621) and 101 (*r*, 0.459) [39]. In the participating treatment-naïve patients, median platelet counts rose during ERT from 63.0 × 10^9^/L at baseline (*n* = 22) to 146.5 × 109/L at week 209 (*n* = 10), whereas the median spleen volume decreased from 16.6 MN at baseline (*n* = 22) to 4.2 multiples of normal at week 209 (*n* = 12) [39]. The ERT-induced reduction in the plasma lyso-Gb1 concentration anteceded the increase in platelets but not the reduction in spleen volume [39].

In a prospective, single-center, longitudinal, case-control study of 169 patients with type 1 GD receiving ERT or SRT, plasma lyso-Gb1 concentration correlated significantly with indicators of severity of visceral disease: splenic volume (*r*, 0.27), liver (*r*, 0.28), and age (*r*, −0.22) [47].

In a prospective, multicenter, cross-sectional, case-control study, lyso-Gb1 content in RBCs was higher in untreated patients with type 1 and neuronopathic GD (0.69 p/mol per kg protein) than in healthy controls (0.15 p/mol per kg protein) and in patients who had been receiving ERT for at least 1 year (0.34 p/mol per kg protein) [40]. Higher concentrations of lyso-Gb1 in RBC membranes correlated with low Hb and abnormal deformability and morphology [40]. Urine lyso-Gb1 concentration correlated with liver volume in a prospective, single-center, cross-sectional study of patients with type 1 GD receiving ERT and healthy controls [44].

Correlations between lyso-Gb1 concentrations and other established biomarkers of GD were evaluated in six studies [33,40,41,44,47,52] (Table S6). Significant correlations between lyso-Gb1 and chitotriosidase concentrations in plasma and RBCs were observed in four studies [33,41,47,52], with Pearson coefficient values from 0.59 to >0.9, and a similar trend reported in one additional study [40]. Correlations between lyso-Gb1 and CCL18 concentrations were reported in three studies [33,40,48]. No correlation between urinary lyso-Gb1 and either chitotriosidase or CCL18 was observed in one study [44].

## 3. Discussion

By systematically evaluating preclinical and clinical publications relevant to lyso-Gb1 in GD up to March 2019, we have identified strong evidence for the use of lyso-Gb1 as a pathogenic mediator of disease and a diagnostic and pharmacodynamic biomarker, and some evidence for the use of lyso-Gb1 as a prognostic and disease-monitoring biomarker in GD. Although lyso-Gb1, chitotriosidase, and CCL18 correlate well with each other in patients with GD, lyso-Gb1 is more sensitive and specific than the other two biomarkers, irrespective of the chitotriosidase genotype [41,50]. Lyso-Gb1 fulfils many of the criteria required for a biomarker in that it is accessible in a clinical sample, easily and reliably quantifiable [33], diagnostically highly valuable, and reflects responses to therapeutic interventions [11]. Further research is warranted with respect to the relationship of lyso-Gb1 with clinical manifestations, including the prediction of comorbidities, burden of disease, and clinical outcomes, to fulfil conditions of an ideal biomarker of disease. Overall, the lyso-Gb1 data in GD adds to the body of evidence that the accumulation of corresponding sphingoid bases in other lysosomal storage disorders are pathogenic and diagnostic [7].

Since the literature search for this systematic review was conducted, additional data on the value of lyso-Gb1 as a biomarker in GD have been published. Lyso-Gb1 measured in dried blood spots was found to be highly sensitive and specific for GD in a case-control study conducted in the Russian Federation [118]. Further, the diagnostic laboratory work-up by Fuller et al. [41] showing the capacity of elevated plasma lyso-Gb1 to identify patients with GD with 100% sensitivity and specificity was extended successfully to testing using dried blood spots and, additionally, samples in a prenatal setting [119]. On the basis of a normal lyso-Gb1 reference interval of <0.16 pmol per dried blood spot, all patients with GD were differentiated from control samples and patients with other inherited metabolic disorders [119]. Indeed, Saville et al. report that in utero lyso-Gb1 quantitation (limit of detection, 1 pmol/mg protein) facilitates GD diagnosis in the prenatal setting [119]. Further, Polo et al. reported a significant elevation of lyso-Gb1 in neonatal dried blood spots using a newly developed and validated assay [120]. These findings support the value of measuring lyso-Gb1 in dried blood spots as a means to conduct high-throughput newborn screening, first conducted by Kang et al. in China [3]. Although few countries have newborn screening programs that include GD, high rates of false positives have been reported on the measurement of enzyme activity on dried blood spots [121,122], and second-tier analyses are being introduced. In North Eastern Italy, lyso-Gb1 testing on dried blood spots was found to have a positive predictive value of 100%, with all neonates returning elevated lyso-Gb1 confirmed as true positives for GD [121].

Data from these studies additionally suggest that lyso-Gb1 concentrations measured in dried blood spots have prognostic power [118,119]. Plasma lyso-Gb1 concentrations were found to be significantly higher in patients with neuronopathic GD than in those with non-neuronopathic disease, even in the neonatal period [119]. A neonate diagnosed at 1 day of age (homozygous for N370S), owing to an affected older sibling, had a plasma lyso-Gb1 level of 70 nmol/L compared with 1070–2620 nmol/L for four neuronopathic patients diagnosed at <20 days of age [119]. Lyso-Gb1 measured in dried blood spots also correlated with hepatomegaly and splenomegaly in the Russian study [118]. Another three studies providing information on plasma lyso-Gb1 as a prognostic biomarker were identified. Firstly, in a family in which all members over two generations presented with splenomegaly owing to four distinct *GBA* genotypes, a plasma lyso-Gb1 level spanning more than one order of magnitude correlated well with the presumed pathogenicity of the genotypes and with hepatosplenomegaly, thrombocytopenia, and bone pain [123]. Secondly, moderate-to-strong correlations between plasma lyso-Gb1 and spleen volume, liver volume, and Hb but not platelet count at baseline were detected in eliglustat clinical trials of treatment-naïve adults with type 1 GD [124]. Thirdly, in untreated pediatric and adult patients with GD, plasma lyso-Gb1 correlated with disease load and severity, with the highest concentrations (up to 200-fold elevations) observed in those with the type 2 and type 3 forms [125].

Four recent studies found that lyso-Gb1 measurement in dried blood spots is useful for monitoring patients with GD [126,127,128]. Among 103 untreated adult patients with type 1 GD who were followed up over a median of 20 years, the median lyso-Gb1 in dried blood spots at the last visit was 108.5 ng/mL (normal, <8 ng/mL) [126]. Patients with the R496H/other genotype had the lowest lyso-Gb1 concentrations and patients refusing therapy had the highest concentrations [126]. Lyso-Gb1 concentrations negatively correlated with platelet count in non-splenectomized patients but not with any other GD-related parameter [126]. In a separate study, up to 10 years of lyso-Gb1 data (measured in plasma and dried blood spots) from 292 patients enrolled in a single center participating in the Gaucher Outcome Survey registry were analyzed with respect to treatment outcomes [127]. Most patients had the homozygous N370S genotype and received relatively low doses of ERT [127]. At the time of first lyso-Gb1 assessment, patients on treatment had lower concentrations of plasma lyso-Gb1 than patients who were untreated at that time [127]. Treatment with ERT resulted in further reductions in plasma lyso-Gb1 in most treatment-naïve and previously treated patients [127]. This study demonstrated that routine lyso-Gb1 monitoring using the dried blood spot assay is feasible. A separate retrospective chart review conducted by the same research group found that lyso-Gb1 concentrations were significantly lower in children with mild type 1 GD than in those with severe type 1 GD and, in untreated children, lyso-Gb1 concentrations were inversely correlated with platelet counts [127]. During follow-up, lyso-Gb1 concentration increased in almost 50% of untreated children, more commonly in younger children [127]. A fourth study determined that lyso-Gb1 monitoring has utility in the assessment of loss of therapeutic effect, as indicated by increased lyso-Gb1 concentrations during periods of treatment interruption during a 3-year evaluation period [128]. Dried blood spot-based quantification of lyso-Gb1 concentrations was validated and applied to 19 patients with GD from a single center in Albania. During the ~25-month period of continuous ERT, there was a tendency for lyso-Gb1 values to decrease over time; however, lyso-Gb1 concentrations were found to increase 1.3–3.8-fold (median, 2.16) after treatment interruption. Findings from this study enabled the separation of patients undergoing treatment from patients not currently receiving treatment, with high sensitivity and specificity [128].

The value of plasma lyso-Gb1 as a pharmacodynamic and response biomarker was reported in three recent studies [124,125,127]. Reductions in plasma lyso-Gb1 concentrations after eliglustat treatment of 92% and 84% in phase II and phase III trials, respectively, correlated well with reductions in hepatosplenomegaly and increases in Hb and platelet counts [124]. Lyso-Gb1, as quantified in dried blood spots, was inversely correlated with Hb in children with type 1 GD receiving ERT in a retrospective chart review [127]. The counterintuitive increase in lyso-Gb1 observed in eight of the treated children was interpreted as suboptimal ERT dosing owing to weight gain [127]. Finally, modest plasma lyso-Gb1 elevations from baseline were observed among treated patients with GD, which may have correlated with residual disease activity due to continued bone marrow involvement, and with decreased hematologic and bone density values; however, in those with advanced disease including complications such as history of osteonecrosis or gammopathy, no significant correlation was observed [125].

It is interesting to speculate as to why not all measures of disease severity correlated with plasma lyso-Gb1 [33]. The generation of lyso-Gb1 in plasma is dependent on GBA activity, Gb1 substrate concentration, acid ceramidase activity, GBA2 activity [45], and transport capacity from the lysosome to cytoplasm to the extracellular space. The role of lyso-Gb1 in specific GD pathologies such as bone and central nervous system disease can be restricted to those anatomical structures and therefore may not manifest as an abnormal level in plasma. In treated patients, it is also possible that SRT and ERT have different effects on lyso-Gb1 dynamics in plasma.

Lyso-Gb1 measurement techniques continued to advance during the conduct of our review. Elevated concentrations of lyso-Gb1 could be detected in only 20 µL of plasma collected from patients with GD using reverse-phase LC-Differential Mobility Spectrometry-MS/MS, which enables the resolution of the corresponding stereoisomer [129]. Compared with plasma analysis, the measurement of lyso-Gb1 in dried blood spots offers a more convenient means of high-throughput testing without the need for an intravenous blood draw, with applications as a companion diagnostic and monitoring tool [119,120,127,130]. Dried-blood spot analysis has the advantage of requiring a smaller volume of blood, and being easily transportable to laboratory facilities, although the concentration of target analyte can potentially be low, necessitating a sensitive and specific assay for detection and quantification [131]. However, results can be influenced by sample integrity, and hematocrit level, which can vary widely, especially in neonates [132]. One assay that requires two 3.2-mm dried blood spot punches and facilitates the measurement of lyso-Gb1 at concentrations as low as 5 ng/mL could differentiate clearly between presumed normal patients and confirmed patients with GD [130]. Another assay demonstrated a high correlation between measurements of lyso-Gb1 concentrations in dried blood spots and plasma in GD; however, the diagnostic performance of lyso-Gb1 in dried blood spots was slightly lower than that in plasma [120].

One important limitation of lyso-Gb1 analyses is the variable methodology employed by different laboratories or within the same laboratory over time, preventing the ability to directly compare findings. In addition, the storage conditions of the pathology samples from which lyso-Gb1 concentrations were measured cannot be assured in some early studies. High variability of longitudinal lyso-Gb1 measurements has been reported in multiple studies, with potential explanations including the impact of the circadian rhythm, effects of nutrition and/or physical activity, or effects of coexisting pathological conditions [128]. Thus, the interpretation of lyso-Gb1 concentrations across studies should focus on trends rather than absolute quantitation. Further, the risk of bias for the different studies and study designs was variable. A risk of bias was particularly high in nRCTs and observational studies (63%) and these should be interpreted with care. Collaboration between laboratories is now required to allow future comparison of inter-laboratory lyso-Gb1 data. Firstly, standardization of the unit of lyso-Gb1 measurement is required across laboratories, as, currently, levels may be expressed in ng/mL, or using SI and ERNDIM units in nmol/L. Cross-validation between laboratories is required to confirm the validity of lyso-Gb1 biomarker data, while quality assurance programs to ascertain that laboratories maintain high levels of statistical reliability when measuring lyso-Gb1 will aid the cross-validation process.

## 4. Methods

The systematic literature review was conducted and reported in line with criteria stipulated by the Preferred Items for Systematic Review and Meta-Analyses Protocols (PRISMA-P) recommendations [133].

### 4.1. Search Strategy

Search terms were developed on the basis of Medical Subject Headings (MeSH), and free-text words and abbreviations related to lyso-Gb1. The specific search terms and Boolean strategy were as follows: (glucosylsphingosine OR GlcSph OR lyso-GL1 OR Lyso-Gb1 OR psychosine) AND Gaucher. The databases searched were the US National Library of Medicine (includes PubMed and MEDLINE), Embase, ScienceOpen, Science.gov, Biological Abstracts, and Sci-Hub. The searches were conducted between February and March 2019, with no restrictions with respect to the search period. Additional references identified from the reference lists of published articles were identified.

### 4.2. Eligibility Criteria

Eligible articles included original research articles (preclinical and clinical) reporting information on lyso-Gb1 in GD published at any time in the medical literature. Meta-analyses were included but systematic (without meta-analysis) or narrative reviews and single-patient case studies were excluded.

### 4.3. Screening and Data Collection

Citations and abstracts retrieved from the searches were compiled to create a single list of references. Abstracts were reviewed by three researchers to determine the articles to be included. If information in the abstract was not sufficient to make a decision, the full text publication was obtained and reviewed. Data were extracted from each eligible publication using a standardized Microsoft Excel^®^ form developed for the systematic review. Extracted data included those related to study design, sample (size, type, and source), lyso-Gb1 assay method, interventions, and type of information on lyso-Gb1 as a pathogenic mediator and biomarker in GD.

### 4.4. Data Analysis

A two-tiered approach to data analyses was applied on the basis of the US Food and Drug Administration/National Institutes of Health Biomarker Working Group definition of a biomarker as follows: “a defined characteristic that is measured as an indicator of normal biological processes, pathogenic processes, or responses to an exposure or intervention, including therapeutic interventions” [12]. The two main categories were as follows: (1) lyso-Gb1 involvement in the pathophysiology of GD, and (2) evidence on lyso-Gb1 as a biomarker in GD. Pathology studies included human autopsy data and preclinical studies reporting on the accumulation and effects of lyso-Gb1. The experimental effects of lyso-Gb1 were graded by the criteria of causation, with controlled studies demonstrating induction (e.g., dose response) and reversibility given more credence than studies showing incidental observations. Biomarker studies were clinical studies reporting on lyso-Gb1 concentrations in subjects with and without GD, and in treated and untreated patients with GD. These data provided evidence for lyso-Gb1 as a potential diagnostic, prognostic, and disease/treatment monitoring biomarker in GD. More credence was given to studies specifically designed to examine lyso-Gb1 as a biomarker in GD than studies reporting observations incidental to lyso-Gb1 levels.

### 4.5. Quality (Risk of Bias) Assessment

A validated tool matched to the study type was used to assess the strength and validity of the empirical data for each individual study. The assessment of the quality of randomized controlled trials was carried out using recommendations from the National Institute for Health and Care Excellence (NICE) single technology appraisal (STA) manufacturer’s template [134]. The Newcastle–Ottawa instrument [135] was used to assess the quality of all non-randomized and observational studies. Animal studies were assessed using the Systematic Review Centre for Laboratory animal Experimentation (SYRCLE) risk of bias tool [136]. Some preclinical studies were not evaluated beyond the recognition of standard scientific methods, as suitable tools were not available at the time of analysis.

## 5. Conclusions

After systematically searching for and evaluating an extensive list of manuscripts published since 1974 according to PRISMA-P guidelines, we conclude that circulating lyso-Gb1 is a selective and sensitive biomarker of GD throughout the course of the disease, as a companion diagnostic and pharmacodynamic biomarker. There is a high degree of confidence that lyso-Gb1 mediates GD pathophysiology but further study is required on its specific roles in this process. Although there is some evidence supporting a role of lyso-Gb1 as a prognostic and disease-monitoring biomarker in GD, the associations of lyso-Gb1 level with prognosis, secondary clinical events, and bone disease requires further research.

## Figures and Tables

**Table 1:** Autopsy and biopsy studies reporting on lyso-Gb1 levels in Gaucher disease (GD).

**Author(s), Year (Reference)**	**Study Design**	**Sample: GD Variant (*n*)**	**Lyso-Gb1 Assay Method**	**Lyso-Gb1 Level**	**Key Finding**
Raghavan et al., 1974 [93]	Retrospective	Spleen: type 1 (*n* = 2)	LC and GLC analysis	5.3 µmol/100 g wet tissue ^a^	First time lyso-Gb1 isolated from GD spleen
Nilsson et al., 1982 [55]	Prospective	Spleen: type 1 (*n* = 4), type 2 (*n* = 3), type 3 (*n* = 12). Liver: type 2 (*n* = 3), type 3 (*n* = 9)	GLC and MS	Spleen (mmol/kg): type 1, 0.07; type 2, 0.16; type 3, 0.19. Liver (mmol/kg): type 2, 0.09; splenectomized type 3, 0.16; non-splenectomized type 3, 0.06	High lyso-Gb1 concentrations were deemed a contributing factor behind commonly seen visceral pathology in patients with GD
Nilsson and Svennerholm, 1982 [56]	Retrospective, case control	Cerebrum/cerebellum ^b^: type 2 (*n* = 5), type 3 (*n* = 8), type unconfirmed [1 or 3] (*n* = 1), control (*n* = 20)	LC and densitometry	Type 2 had the highest level (4–12 µmol/kg), some 2 to 3 orders of magnitude higher than in control brain	Lyso-Gb1, never detected in normal human brain, was demonstrated at high levels in brains from all patients with GD
Conradi et al., 1984 [62]	Retrospective	Cerebral cortex: type 3 (*n* = 5)	HPTLC	0.3–6.3 µmol/kg versus undetectable levels in controls	Highest lyso-Gb1 concentrations seen in cases with the most advanced nerve cell loss
Nilsson et al., 1985 [54]	Retrospective	Liver and brain: type 1 (*n* = 2), control (*n* = 5)	TLC and densitometry	Patient 1 (µmol/kg wet weight): spleen, 0.16; liver, 0.10; cerebral cortex, 2.4; cerebellar cortex, 1.7. Patient 2 (µmol/kg wet weight): spleen, 0.14; liver, 0.04; cerebral cortex, 0.2; cerebellar cortex, 0.06. Controls: undetectable	Hepatic lyso-Gb1 2-fold greater in a severely affected 3-year-old American Black patient compared with a 56-year-old Ashkenazi Jewish patient. Lyso-Gb1 was found in large amounts only in cerebral and cerebellar cortices from the severely affected patient
Orvisky et al., 2000 [58]	Retrospective, case control	Fetal tissue (brain, liver, and spleen): type 2 (*n* = 2), control (*n* = 3)	LC and HPLC then fluorescence quantitation	Spleen: 190 ng/mg protein; liver: 92–114 ng/mg protein ^c^; brain: 305–437 ng/mg protein ^c^. Control samples: <0.3 ng/mg protein	Lyso-Gb1 was elevated relative to human control samples
Orvisky et al., 2002 [57]	Prospective, case control	Spleen: type 1 (*n* = 8), type 2 (*n* = 4), type 3 (*n* = 4). Brain: type 1 (*n* = 1), type 2 (*n* = 8), type 3 (*n* = 4). Control (*n* = 9)	HPLC then fluorescence quantitation	Spleen (ng/mg protein): type 1, 54–728; type 2, 133–1200; type 3, 109–1298. Brain (ng/mg protein): type 1, 1.0 (normal); type 2, 24–437; type 3, 14–32. Control samples: 0 ng/mg protein	Lyso-Gb1 accumulation in the brain correlated with CNS involvement but splenic lyso-Gb1 levels bore no relation to the type of GD, the age of the patient, the genotype, or the clinical course
Park et al., 2003 [59]	Retrospective, case control	Brain: type 3 with progressive myoclonic epilepsy (*n* = 2), control (*n* = 9)	HPLC then fluorescence quantitation	Brain (ng/mg protein): type 3, 22 and 32; control: 0.04–1.2	35- to 50-fold increase in brain lyso-Gb1 concentrations observed in two patients with type 3 GD relative to controls
Tayebi et al., 2003 [61]	Retrospective, case control	Brain: type 1 (*n* = 3). Historical controls: type 1 (*n* = 3), type 2 (*n* = 8), type 3 (*n* = 4). Healthy control: (*n* = 9)	HPLC then fluorescence quantitation	Brain: type 1, 0.4–1.3 ng/mg protein. Historical controls (ng/mg protein): type 1, 0.9–1.4; type 2, 24–437; type 3, 14–32. Healthy control: 0.04–1.2 ng/mg protein	Brain lyso-Gb1 concentrations were in the normal range among three patients with type 1 GD
Lloyd-Evans et al., 2003 [63]	Case control	Brain: type 2 (*n* = 1), control (*n* = 1)	Acetylation with ^3^H-acetic anhydride	Brain (ng/mg protein): type 2, 4.88; control, 0	Lyso-Gb1 detected in the type 2 GD brain with no detectable levels in control brain microsomes

^a^: High potential for underestimation owing to isolation procedures required to yield highly purified samples for positive identification and chemical characterization. ^b^: All subjects were juveniles. ^c^: Higher values in infants with a gestational age of 22 weeks than in those with a gestational age of 11 weeks. CNS: central nervous system; GD: Gaucher disease; GLC: gas-liquid chromatography; HPLC: high-performance liquid chromatography; HPTLC: high-performance thin-layer chromatography; LC: liquid chromatography; lyso-Gb1: glucosylsphingosine; MS: mass spectrometry; TLC: thin-layer chromatography.

**Table 2:** In vitro studies reporting association between lyso-Gb1 and GD pathology.

Author(s), Year (Reference)	Sample	Observation	Strength of Association with Lyso-Gb1 ^a^
**Human Cells**			
Hannun et al., 1987 [105]	Mixed micelles and human platelets	Aberrant signal transduction: exogenous lyso-Gb1 inhibited protein kinase C	Induction
Im et al., 2001 [106]	HEK293 cells and RH7777 cells (rat)	Globoid cell formation: exogenous lyso-Gb1 evoked giant multinucleated cell formation via activation of the proton-sensing G protein–coupled receptor T-cell death-associated gene 8	Induction
Schueler et al., 2003 [100]	Cultured human cholinergic neuron-like LA-N-2 cells	Cytotoxicity: exogenous lyso-Gb1 cytotoxic to cultured human cholinergic neuron-like LA-N-2 cells. Partial recovery when cells switched to lyso-Gb1-free medium	Induction
Giri et al., 2006 [104]	M03.13 cell line: Immortal human–human hybrid cell line expressing phenotypic characteristics of primary oligodendrocytes	Pro-inflammatory: lyso-Gb1 induces arachidonic acid release in oligodendrocytes	Induction
Sun et al., 2015 [103]	Neural precursor cells and neurons differentiated from pluripotent stem cells derived from fibroblasts collected from a patient with type 2 GD, a heterozygous carrier (L444P and 1483G > C and 1497G > C), and a control	Electrophysiologic: CBE-treated control neurons had significantly increased lyso-Gb1 and altered physiological properties comparable to those from type 2 GD-derived neurons	Incidental
Nair et al., 2015 [97]	Human and murine type 2 natural killer T cells expressing the T-follicular helper phenotype	Pro-inflammatory: frequency of lyso-Gb1-specific T cells in GD mouse models and patients correlates with disease activity and therapeutic response	Correlative
Aflaki et al., 2016 [108]	Pluripotent stem cell-derived dopaminergic neurons derived from fibroblasts collected from patients with type 1 and 2 GD with Parkinsonism	Cytotoxicity: α-synuclein and lyso-Gb1 elevated in neurons from patients with parkinsonism or type 2 GD. Effect reversed by NCGC607, a small-molecule non-inhibitory chaperone of GBA	Incidental
Smith et al., 2018 [83]	Cultures of human umbilical vein endothelial cells	Cytotoxicity: exogenous lyso-Gb1 concentration-dependent impairment of endothelial cytokinesis	Induction
Reed et al., 2018 [98]	Osteoblasts differentiated from mesenchymal stem cells isolated from bone marrow aspirates of patients with type 1 GD and control subjects	Impaired osteoblasts: exogenous lyso-Gb1 reduced mesenchymal stem cell viability, potential for differentiating into osteoblasts, and reduced calcium deposition of these osteoblasts	Induction
**Animal Cells**			
Taketomi et al., 1976 [107]	Animal RBCs	Cytotoxicity: exogenous lyso-Gb1 lyses RBCs	Induction
Igisu et al., 1988 [96]	Rat liver mitochondria	Metabolic: exogenous lyso-Gb1 inhibited cytochrome c oxidase	Induction
Atsumi et al., 1993 [64]	NIH 3T3 (mouse fibroblast) cells and PC12 (rat neural-crest-derived neoplastic) cells	Cytotoxicity: lyso-Gb1 was directly cytotoxic to both cell lines	Induction
Liu et al., 2012 [74]	*GBA1* gene deletion in hematopoietic and mesenchymal lineages (knockout/LoxP/Mx1)	Immune dysregulation: exogenous lyso-Gb1 inhibited by >50% the proliferation of HSC precursors. Proliferation of GBA-deficient HSCs inhibited by Gb1 and lyso-Gb1	Induction
Xu et al., 2014 [102]	Newborn neural cells from type 3 GD mouse model	Cytotoxicity: amyloid precursor protein/α-synuclein accumulation in neural cells correlated with increased cellular Gb1 and lyso-Gb1 levels	Correlation
Westbroek et al., 2016 [101]	Immortalized cortical neurons from embryonic null allele *GBA*^−/−^ mice and the control littermate (*GBA*^+/+^)	Cellular pathology: lyso-Gb1 accumulation associated with enlarged lysosomes, and an impaired ATP-dependent calcium-influx response	Incidental
Taguchi et al., 2017 [84]	*GBA* mutant (N370S, L444P) and knockout mouse models crossed with an α-synuclein transgenic PD mouse	Cytotoxicity: lyso-Gb1 accumulation promoted α-synuclein aggregation	Induction

^a^: Association graded by causation criteria where “induction” pertains to controlled studies demonstrating dose response and/or reversibility of action, “correlation” pertains to a pattern between a variable and lyso-Gb1 level, and “incidental” pertains to observations that could have been spontaneous or explained by factors other than lyso-Gb1. ATP: adenosine triphosphate; CBE: conduritol B epoxide; GBA: glucosylceramidase; GD: Gaucher disease; HSC: hematopoietic stem cells; lyso-Gb1: glucosylsphingosine; PD: Parkinson disease; RBC: red blood cell.

**Table 3:** In vivo studies reporting association between lyso-Gb1 and GD pathology.

Author(s), Year (Reference)	Sample	Observation	Strength of Association with Lyso-Gb1 ^a^
**Pharmacologically Induced GD**			
Rocha et al., 2015 [81]	Murine: wild-type treated with CBE ^b^ or vehicle	Brain α-synuclein aggregation, region-specific pre-degenerative changes, and neurodegeneration. Microglia and complement C1q activation	Incidental
Marshall et al., 2016 [76]	Murine: wild-type treated with CBE	Ibiglustat^b^ reduced elevated levels of lyso-Gb1 in the liver and brain by >70% and >20%, respectively, relative to controls. Ibiglustat reduced the extent of gliosis and neurobehavioral deficits	Incidental
Vardi et al., 2016 [85]	Murine: wild-type treated with CBE	The average day of death of the mice correlated with Gb1 levels (*r*^2^ = 0.91) and lyso-Gb1 levels (*r*^2^ = 0.83)	Correlation
Lukas et al., 2017 [75]	Murine: wild-type treated with lyso-Gb1	Reduced Hb and hematocrit, increased spleen weights, and a slight inflammatory tissue response	Induction
**D409V Point Mutation**			
Pandey et al., 2017 [78]	Murine: heteroallelicmutations in *GBA1*, a point mutation, and a D409V knockout (*GBA1*^9V/−^)	Lyso-Gb1-specific IgG2a autoantibodies not detected in wild-type and *GBA*1^9V/−^ mice	Cause and lack of effect
**Conditional Type 1 GD**			
Mistry et al., 2010 [77]	*GBA1* gene deletion in hematopoietic and mesenchymal lineages (knockout/LoxP/Mx1)	Hepatosplenomegaly, anemia, thrombocytopenia, and accumulation of storage cells in the liver, spleen, bone marrow, lymph nodes, and thymus. Inhibition of PKC-mediated osteoblast proliferation and early differentiation. PMA-induced precursor proliferation. Splenomegaly correlated with the tissue content of Gb1 and lyso-Gb1	Incidental
Liu et al., 2012 [74]	*GBA1* gene deletion in hematopoietic and mesenchymal lineages (knockout/LoxP/Mx1)	Correlation between splenic lyso-Gb1 levels and splenomegaly (*r*^2^ = 0.48; *p* = 0.00004)	Correlation
Mistry et al., 2014 [45]	Murine: double-mutant Mx1-Cre^+^:GD1:*GBA2*^−^^/^^−^	Despite elevated lyso-Gb1 levels, concomitant deletion of the *GBA2* gene in GD1 mice rescued hepatosplenomegaly, cytopenia, osteopenia, and hypercytokinemia	Incidental
Pavlova et al., 2015 [79]	Murine: *GBA*^tm1Karl/tm1Karl^Tg(Mx1-Cre)1Cgn/0 versus induced *GBA*^tm1Karl/tm1Karl^ and *GBA*^tm1Karl/+^ genotypes	Elevated lyso-Gb1 was associated with occurrence of B-cell lymphomas and monoclonal gammopathy	Incidental
**Neuronal GD**			
Cabrera-Salazar et al., 2010 [89]	Murine: K-14Cre^+^ GBA^lnl/lnl^ and wild-type	Intracerebroventricular administration of recombinant human GBA produced dose-dependent reductions in brain lyso-Gb1 level and improved survival	Correlation
Cabrera-Salazar et al., 2012 [90]	Murine: K-14Cre^+^ GBA^lnl/lnl^ and wild-type	Intraperitoneal administration of a glucosylceramide synthase inhibitor reduced brain lyso-Gb1 level and improved survival	Incidental
Farfel-Becker et al., 2013 [70]	Neuronopathic GD murine: *GBA*^flox/flox^; nestin-Cre	No neuronal loss	Correlation
Smith et al., 2018 [83]	Type 2 murine: K-14Cre^+^ *GBA*^lnl/lnl^ versus wild-type	Diminished cerebral microvascular density	Incidental
Dasgupta et al., 2015 [68]	Type 3 (subacute) murine: transgenic 4L;C*^h^	Activated microglial cells, reduced number of neurons, and aberrant mitochondrial function in the brain followed by deterioration in motor function	Incidental
Dai et al., 2016 [67]	Type 3 (chronic) murine: D409V and null alleles (9V/null)	α-synuclein aggregation and hepatosplenomegaly	Incidental
Marshall et al., 2016 [76]	Type 3 (subacute) murine: transgenic 4L;C*^h^	Ibiglustat reduced elevated levels of lyso-Gb1 in the liver and brain by >40%, and also reduced the extent of gliosis and paresis. Ibiglustat-treated 4L;C* mice had a ~30% increase in lifespan	Incidental
Sardi et al., 2017 [82]	Type 3 (chronic) murine: D409V/D409 alleles (9V/9V)	Pathologies associated with α-synuclein aggregation	Incidental

^a^: Association graded by causation criteria where “induction” pertains to controlled studies demonstrating dose response and/or reversibility of action, “correlation” pertains to a pattern between a variable and lyso-Gb1 level, and “incidental” pertains to observations that could have been spontaneous or explained by factors other than lyso-Gb1. ^b^: Specific GBA inhibitors. CBE: conduritol B epoxide; GBA: glucosylceramidase; Hb: hemoglobin; lyso-Gb1: glucosylsphingosine; *r*^2^: Pearson product moment correlation coefficient; PKC: protein kinase C; PMA: phorbol 12-myristate 13-acetate (PKC activator).

**Table 4:** Cross-sectional, observational studies reporting on lyso-Gb1, as measured by liquid chromatography techniques, in subjects with and without GD.

Author(s), Year (Reference)	Study Design	Population Type	Key Diagnostic Finding
**Incidental Studies**			
Moraitou et al., 2014 [46]	Prospective, 2 centers	Type 1 GD (*n* = 24), type 2 GD (*n* = 3), healthy controls (*n* = 13)	Plasma lyso-Gb1 concentrations were elevated >200-fold in patients with type 1 GD relative to controls
Mirzaian et al., 2015 [44]	Prospective, single center	Type 1 untreated GD (*n* = 55), healthy controls (*n* = 53)	Plasma (median, 230.7 versus 1.3 nM) and urine (median, 1.20 versus 0.01 nM) lyso-Gb1 concentrations were elevated in untreated symptomatic patients with type 1 GD relative to controls
Ferraz et al., 2016 ^b^ [26]	Prospective, single center	Symptomatic type 1 GD (*n* = 69), healthy controls (*n* = 79)	Plasma lyso-Gb1 concentrations were elevated 300-fold in symptomatic patients with type 1 GD relative to controls
Franco et al., 2017 [40]	Prospective, multicenter	Type 1 GD and neuronopathic (*n* = 16)	The RBC membrane lyso-Gb1 concentration in untreated patients with type 1 GD was increased relative to healthy controls (median, 0.69 versus 0.15)
Kang et al., 2017 [3]	Prospective, single center	Mixed GD types (*n* = 9)	Plasma lyso-Gb1 concentrations were 266 ng/mL in eight patients with type 1 GD and 4.7 ng/mL in one child with GD (normal range, 0.17–1.18 ng/mL)
**Diagnostic Studies Reporting Lyso-Gb1 as a Primary Endpoint**			
Dekker et al., 2011 [33]	Prospective, multicenter	Type 1 GD (*n* = 64), GD carriers (*n* = 34). Healthy age-matched controls (*n* = 28)	Plasma lyso-Gb1 concentrations were elevated >200-fold in patients with type 1 GD relative to controls (median, 231 versus 1.3 nM)
Rolfs et al., 2013 [50]	Retrospective, single center	Mixed GD (*n* = 129), ^b^ GD carriers (*n* = 13), healthy controls (*n* = 148), other LSDs (*n* = 261) ^a,c^	Patients with GD displayed elevated plasma concentrations of lyso-Gb1 >12 ng/mL whereas the comparison control groups revealed concentrations below this pathological cut-off. The 12 ng/mL cut-off value had 100% sensitivity and specificity
Fuller et al., 2015 [41]	Prospective, single center	Mixed GD (*n* = 15), healthy adults (*n* = 50), no metabolic disorders (*n* = 1350), other metabolic disorders (*n* = 49)	Plasma lyso-Gb1 concentrations were elevated in patients with GD (median, 920 pmol/mL) compared with unaffected controls and patients with 16 other metabolic disorders (median, ≤9 pmol/mL)
Murugesan et al., 2016 [47]	Prospective	Untreated type 1 GD (*n* = 169), healthy controls (*n* = 41)	Plasma lyso-Gb1 concentrations were elevated >200-fold in patients with type 1 GD relative to controls (181 versus 1.5 ng/mL). A 4 ng/mL cut-off value had 100% sensitivity and specificity
Chipeaux et al., 2017 [38]	Prospective, multicenter	Type 1 GD (*n* = 15)^d^	Lyso-Gb1 was one to two orders of magnitude higher in both plasma and RBCs of patients with GD compared with healthy controls. Lyso-Gb1 was a more powerful biomarker than Gb1, sphingosine, and sphingosine-1-phosphate

^a^: All subjects were non-Jewish and Caucasian. ^b^: A total of 98 patients had genetically diagnosed GD. ^c^: The GD patient group and healthy control group were younger than the obligate carriers and patients with other LSDs. ^d^: All patients were splenectomized and untreated. GD: Gaucher disease; LSD: lysosomal storage disorder; lyso-Gb1: glucosylsphingosine; MS: mass spectrometry; RBC: red blood cell.

**Table 5:** Overview of studies assessing lyso-Gb1, as measured by liquid chromatography tandem mass spectrometry (LC/MS/MS), as a prognostic biomarker in GD.

Author(s), Year (Reference)	Study Design	Population Type	Key Prognostic Findings
Dekker et al., 2011 [33]	Prospective, multicenter, cross-sectional, observational	Type 1 GD (*n* = 64), GD carriers (*n* = 34), healthy controls (*n* = 28)	Plasma lyso-Gb1 concentrations were lower among N370S *GBA* homozygous individuals than N370S/L444P *GBA* patients. Only within the group of N370S *GBA* homozygous patients was a clear relation between disease severity and plasma lyso-Gb1 concentrations observed. ^a^ There was a positive correlation between plasma lyso-Gb1 and liver volume, and a negative correlation with bone marrow fat fraction
Rolfs et al., 2013 [50]	Retrospective, single center, observational	Mixed GD (*n* = 129), GD carriers (*n* = 13), healthy controls (*n* = 148), other LSDs (*n* = 261) ^b^	There were correlations between plasma lyso-Gb1 concentrations and the *GBA* mutations N370S and L444P. Mutation L444P was associated with higher plasma lyso-Gb1 (median, 185 ng/mL) than the milder N370S mutation (median, 143 ng/mL). Plasma lyso-Gb1 concentrations were higher in homozygous (N370S/N370S, 143 ng/mL; L444P/L444P, 185 ng/mL) than in compound heterozygous (N370S, 77 ng/mL; L444P, 107 ng/mL) GD mutations
Ferraz et al., 2016^b^ [26]	Prospective, single center, cross-sectional, observational	Type 1 GD (*n* = 69), controls (*n* = 79)	Lyso-Gb1 concentrations were excessive in cultured fibroblasts of a collodion patient with GD (homozygous for the recombination RecNci allele) with virtually no residual GBA activity versus more modest elevations among patients with type 1 GD and neuronopathic variants
Elstein et al., 2017 [39]	Exploratory pooled analysis of phase 3 clinical trials	Type 1 GD (*n* = 22)	Mean plasma lyso-Gb1 concentrations were higher for patients with ≥ 1 allele with the N370S mutation (N370S/N370S or N370S/other, 364 ng/mL; *n* = 17) than for patients with non-N370S mutations (185 ng/mL; *n* = 5)

^a^: Clinical severity assessment at baseline was performed using a severity scoring index [116]. ^b^: All subjects were non-Jewish and caucasian. GBA: glucosylceramidase; GD: Gaucher disease; LSD: lysosomal storage disorder; lyso-Gb1: glucosylsphingosine.

**Table 6:** Clinical studies reporting an association between reduced lyso-Gb1 concentration and outcomes.

Author(s), Year (Reference)	Study Design	Population Type	Treatment	Observation	Strength of Association ^a^
Narita et al., 2016 [49]	Clinical trial, multicenter, open-label, pilot	Neuronopathic GD (*n* = 5), healthy controls (*n* = 37)	ERT plus ambroxol ^b^	As CSF lyso-Gb1 concentrations decreased, myoclonus, seizures, and pupillary light reflex dysfunction markedly improved	Incidental
Smid et al., 2016 [51]	Observational, retrospective, longitudinal	Type 1 GD, treatment-naïve, and ERT experienced (*n* = 17)	ERT (*n* = 4); SRT: eliglustat (*n* = 6) or miglustat (*n* = 9)	As plasma lyso-Gb1 concentrations decreased, platelet counts, Hb, and bone marrow fat fraction increased or stabilized whereas spleen and liver volumes decreased or stabilized	Incidental
Mistry et al., 2017 [36]	Clinical trial, phase 3, randomized, multicenter, placebo-controlled, crossover	Type 1 GD and treatment-naïve (*n* = 40)	SRT: eliglustat	As plasma lyso-Gb1 concentration decreased, platelet counts and Hb increased whereas spleen and liver volumes decreased. Continued eliglustat for 9 more months resulted in incremental improvement of all disease parameters	Incidental
Arkadir et al., 2018 [37]	Observational, retrospective, multicenter, longitudinal	Type 1 GD, non-splenectomized, and N370S homozygotes (*n* = 20)	ERT: imiglucerase (*n* = 4), taliglucerase alfa (*n* = 4), or velaglucerase alfa (*n* = 17)	As plasma lyso-Gb1 concentrations decreased, platelet counts and Hb increased whereas spleen volume decreased from baseline	Incidental
Lukina et al., 2019 [43]	Clinical trial, phase 2, multicenter	Type 1 GD and treatment-naïve (*n* = 26)	SRT: eliglustat for 8 years	As plasma lyso-Gb1 concentrations decreased, platelet counts and Hb increased whereas spleen and liver volumes decreased from baseline. Lumbar spine T-score increased to normal range from baseline	Incidental
Mirzaian et al., 2015 [44]	Observational: prospective, single center, case control, cross-sectional	Type 1 GD (*n* = 55), healthy controls (*n* = 53)	ERT	Urine lyso-Gb1 concentration correlated with liver volume	Correlation
Murugesan et al., 2016 [47]	Observational, prospective, single-center, longitudinal, case control	Type 1 GD (*n* = 169), healthy controls (*n* = 41)	ERT (*n* = 155); SRT: eliglustat (*n* = 14)	Plasma lyso-Gb1 concentration correlated with hepatomegaly, splenomegaly, and splenectomy	Correlation
Chipeaux et al., 2017 [38]	Observational, prospective, multicenter, cross-sectional, case control	Type 1 GD, treatment-naïve, and splenectomized (*n* = 15); controls (*n* = 11)	None	Lyso-Gb1 concentrations in plasma and RBC membranes correlated inversely with Hct	Correlation
Franco et al., 2017 [40]	Observational, prospective, multicenter, cross-sectional, case control	Type 1 GD and neuronopathic (*n* = 31)	ERT (*n* = 15); untreated (*n* = 16)	Lyso-Gb1 content in GD RBCs correlated with low Hb levels. There were correlations between lyso-Gb1 overload in GD RBC membranes and abnormal deformability and morphology	Correlation
Elstein et al., 2017 [39]	Exploratory pooled analysis of phase 3 clinical trials	Type 1 GD and treatment-naïve (*n* = 22); ERT switch (*n* = 21)	ERT: velaglucerase alfa	Treatment-naïve patients: decreasing plasma lyso-Gb1 concentrations correlated with increasing platelet counts and decreasing spleen volumes. Patients who switched: there was a moderate correlation between plasma lyso-Gb1 and platelet counts	Correlation

^a^: Association graded by causation criteria where “correlation” pertains to a pattern between a variable and lyso-Gb1, and “incidental” pertains to observations that could have been spontaneous or explained by factors other than lyso-Gb1. ^b^: Enhances endogenous GBA activity in the murine central nervous system [117]. CSF: cerebrospinal fluid; ERT: enzyme replacement therapy; GD: Gaucher disease; Hb: hemoglobin; Hct: hematocrit; lyso-Gb1: glucosylsphingosine; RBC: red blood cell; SRT: substrate reduction therapy.

## Supplementary Materials

The following are available online at https://www.mdpi.com/1422-0067/21/19/7159/s1. Table S1: Quality assessment of RCTs using National Institute for Health and Care Excellence (NICE) single technology appraisal (STA). Table S2: Quality assessment of nRCTs using the Newcastle-Ottawa scoring (NOS) tool. Table S3: Quality assessment of animal studies using Syrcle. Table S4: In vitro studies reporting on intracellular lyso-Gb1 levels in GD models or cell lines. Table S5: In vivo studies reporting on lyso-Gb1 accumulation in GD models. Table S6: Clinical studies reporting on lyso-Gb1 levels, as measured by liquid chromatography techniques, in GD during treatment with ERT and/or SRT.

## Abbreviations

AMRF	Action myoclonus-renal failure
CBE	Conduritol B epoxide
CCL18	(C-C motif) ligand 18
CNS	Central nervous system
CSF	Cerebrospinal fluid
ERT	Enzyme replacement therapy
GBA	Glucosylceramidase
GD	Gaucher disease
Hb	Hemoglobin
HPLC	High-performance liquid chromatography
HPTLC	High-performance thin-layer chromatography
LC/MS/MS	Liquid chromatography tandem mass spectrometry
Lyso-Gb1	Glucosylsphingosine
NICE	National Institute for Health and Care Excellence
PRISMA-P	Preferred Items for Systematic Review and Meta-Analyses Protocols
RBC	Red blood cell
SRT	Substrate reduction therapy
STA	Single technology appraisal
SYRCLE	Systematic Review Centre for Laboratory animal Experimentation
TDAG8	T cell death-associated gene 8
TLC	Thin-layer chromatography.
