# Supplementary material for: Value of Glucosylsphingosine (Lyso-Gb1) as a Biomarker in Gaucher Disease: A Systematic Literature Review
Source: Int J Mol Sci. 2020 Sep 28;21(19):7159. doi: 10.3390/ijms21197159 (PMC7584006; doi:10.3390/ijms21197159)
Supplement: Supplementary file 1 [file ijms-21-07159-s001.pdf]

Supplementary materials

Table S1: Quality assessment of RCTs using National Institute for Health and Care Excellence (NICE) single technology appraisal (STA)

Author	Year	Randomization	Concealment of Allocation	Groups Similar at Baseline	Blinded to Allocation	Drop Out Imbalance?	More Outcomes than Reported	ITT?
		Grade	Grade	Grade	Grade	Grade	Grade	Grade
Mistry	2017	Low risk	Unclear	Unclear	Low risk	Low risk	Low risk	Low risk

Table S2. Quality assessment of nRCTs using the Newcastle-Ottawa scoring (NOS) tool

Author	Year	Selection				Comparability	Outcome			Total score
		Representativeness of the exposed cohort	Selection of the non-exposed cohort	Ascertainment of exposure	Outcome of interest not present at start of study	Comparability of cohorts on the basis of the design or analysis	Assessment of outcome	Was follow-up long enough for outcomes to occur	Adequacy of follow up of cohorts	
Arkadir	2018	1	0	1	0	0	1	1	1	5
Chipeaux	2017	1	1	1	0	0	0	1	1	5
Conradi	1984	0	0	1	0	0	1	1	1	4
Dekker	2011	1	0	1	0	1	0	1	0	4
Elstein	2017	1	0	1	0	0	0	1	0	3
Ferraz	2016b	0	0	1	0	0	0	1	0	2
Franco	2017	1	0	0	1	1	0	1	0	4
Fuller	2015	1	1	1	0	0	1	1	1	6
Gaspar	2014	0	0	1	0	0	0	1	0	2
Kang	2017	1	1	0	0	0	0	1	0	3
Lloyd-Evans	2003	0	0	1	0	0	0	1	0	2
Lukina	2019	1	0	1	0	0	1	1	1	5
Mirzaian	2015	1	1	1	1	1	1	1	0	7
Mistry	2014	0	0	0	0	0	0	0	0	0
Moraitou	2014	1	1	0	0	0	0	1	0	3
Murugesan	2016	1	0	1	0	0	0	1	1	4
Murugesan	2018	1	0	1	0	1	0	1	0	4

Narita	2016	0	0	1	0	0	1	1	0	3
Nilsson	1982	1	0	1	0	0	0	0	0	2
Nilsson	1985	0	0	1	0	0	0	1	1	3
Nilsson and Svennerholm	1982	0	0	1	0	0	0	0	0	1
Orvisky	2000	0	0	0	0	0	0	0	0	0
Orvisky	2002	1	0	1	0	0	0	0	0	2
Park	2003	1	0	1	0	0	0	0	1	3
Raghavan	1974	0	0	0	0	0	0	0	0	0
Rolfs	2013	1	1	1	0	1	0	1	1	6
Smid	2016	1	0	1	0	0	0	1	1	4
Tayebi	2003	1	0	1	0	0	0	0	1	3
Tylki-Szymanska	2018	1	0	1	0	1	0	0	0	3
Zhang	2017	1	0	1	0	0	0	0	0	2

Table S3. Quality assessment of animal studies using Syrcle

Author	Year	Allocation Sequence	Groups Similar at Baseline	Concealment	Random Housing	Caregivers/ Investigators Blinded	Random Assessment	Outcome Assessor Blinded	Incomplete Data	Free of Selective Outcome Reporting	Free of Other Problems?
Atsumi	1993	Unclear	Low risk	Unclear	Low risk	Unclear	Unclear	Unclear	Low risk	Low risk	Unclear
Barnes	2014	Unclear	Low risk	Low risk	Low risk	Unclear	Unclear	Unclear	Low risk	Low risk	Low risk
Bodennec	2003	Unclear	NA	NA	Low risk	Unclear	Unclear	Unclear	Low risk	Low risk	Low risk
Cabrera-Salazar	2010	Unclear	Low risk	Unclear	Low risk	Unclear	Unclear	Unclear	Low risk	Low risk	Low risk
Cabrera-Salazar	2012	Unclear	Low risk	Unclear	Low risk	Unclear	Unclear	Unclear	Low risk	Low risk	High Risk
Dahl	2015	Unclear	Low risk	Unclear	Low risk	Unclear	Unclear	Unclear	Low risk	Low risk	Low risk
Dai	2016	Unclear	Low risk	Unclear	Low risk	Unclear	Unclear	Unclear	Low risk	Low risk	Low risk
Dasgupta	2015	Unclear	Low risk	Unclear	Unclear	Unclear	Unclear	Unclear	Low risk	Low risk	Low risk
Enquist	2007	Unclear	Low risk	Unclear	Low risk	Unclear	Unclear	Unclear	Low risk	Low risk	Low risk
Farfel-Becker	2013	Unclear	Low risk	Unclear	Unclear	Unclear	Unclear	Unclear	Low risk	Low risk	Low risk
Ferraz	2016a	Unclear	Low risk	Unclear	Low risk	Unclear	Unclear	Unclear	Low risk	Low risk	Low risk
Hamler	2017	Unclear	Low risk	Unclear	Unclear	Unclear	Unclear	Unclear	Low risk	Low risk	High Risk
Karageorgos	2016	Unclear	Low risk	Unclear	Low risk	Unclear	High risk	High risk	Low risk	Low risk	Low risk
Liu	2012	Unclear	Low risk	Unclear	Low risk	Unclear	Unclear	Unclear	Low risk	Low risk	Unclear
Lukas	2017	Unclear	Low risk	Unclear	Low risk	Unclear	Unclear	Unclear	Low risk	Low risk	High risk
Marshall 2016	2016	Unclear	Low risk	Unclear	Low risk	Unclear	Unclear	Unclear	Low risk	Low risk	High risk
Mistry	2010	Unclear	Low risk	Unclear	Low risk	Unclear	Unclear	Unclear	Low risk	Low risk	High Risk
Mistry	2014	Unclear	Low risk	Unclear	Low risk	Unclear	Unclear	Unclear	Low risk	Low risk	High Risk

Orvisky	2000	Unclear	Low risk	Unclear	Low risk	Unclear	Unclear	Unclear	Low risk	Low risk	Low risk
Pandey	2017	Unclear	Low risk	Unclear	Low risk	Unclear	Unclear	Unclear	Low risk	Low risk	Low risk
Pavlova	2013	Unclear	Low risk	Unclear	Low risk	Unclear	Unclear	Unclear	Low risk	Low risk	Low risk
Pavlova	2015	Unclear	NA	NA	Low risk	Unclear	Unclear	Unclear	Low risk	Low risk	Low risk
Rocha	2015	Unclear	NA	NA	Low risk	Unclear	Unclear	Unclear	Low risk	Low risk	High Risk
Sardi	2017	Unclear	Low risk	Unclear	Low risk	Unclear	Unclear	Unclear	Low risk	Low risk	High Risk
Smith	2018	Unclear	Low risk	Unclear	Low risk	Unclear	Unclear	Unclear	Low risk	Low risk	Low risk
Sun	2010	Unclear	Low risk	Unclear	Low risk	Unclear	Unclear	Unclear	Low risk	Low risk	Low risk
Sun	2011	Unclear	Low risk	Unclear	Low risk	Unclear	Unclear	Unclear	Low risk	Low risk	Low risk
Sun	2012	Unclear	Low risk	Unclear	Low risk	Unclear	Unclear	Unclear	Low risk	Low risk	Low risk
Sun	2013	Unclear	Low risk	Unclear	Low risk	Unclear	Unclear	Unclear	Low risk	Low risk	Low risk
Taguchi	2017	Unclear	Low risk	Unclear	Low risk	Unclear	Unclear	Unclear	Low risk	Low risk	High risk
Vardi	2016	Unclear	Low risk	Unclear	Low risk	Unclear	Unclear	Unclear	Low risk	Low risk	Low risk
Zhang	2017	Unclear	Low risk	Unclear	Low risk	Unclear	Unclear	Unclear	Low risk	Low risk	Unclear

Table S4. In vitro studies reporting on intracellular lyso-Gb1 levels in GD models or cell lines.

Author(s), Year [Reference]	Sample	Intervention	Key Findings
**Human**			
Sasagasako et al., 1994 [1]	Cultured fibroblasts from patients with types 1, 2, and 3 GD and controls	GD cells exposed to lyso-Gb1 loading. Controls exposed to lyso-Gb1 loading and CBE (a specific GBA inhibitor)	Lyso-Gb1 was not detected in GD cells but accumulated in control cells exposed to CBE. After lipid loading, lyso-Gb1 degradation in GD cells was similar to that in control cells
Aflaki et al., 2014 [2]	Macrophages from patients with types 1 and 2 GD and controls	NCGC00188758, (a non-inhibitory chaperone of GBA)	Type 2 GD primary macrophages had an 18-fold higher level of lyso-Gb1 than did control clonal macrophages, whereas primary macrophages and clonal macrophages with the N370S/N370S genotype had a 2.8- and 3.2-fold higher level than did controls, respectively. CGC00188758 reduced lyso-Gb1 concentrations in all cell types tested
Sun et al., 2015 [3]	Neural precursor cells and neurons differentiated from pluripotent stem cells derived from fibroblasts collected from a patient with type 2 GD, a heterozygous carrier, and a control	CBE	Lyso-Gb1 concentrations significantly increased in all untreated type 2 cells. CBE-treated control neurons had increased Gb1 and lyso-Gb1 levels
Aflaki et al., 2016 [4]	Pluripotent stem cell–derived dopaminergic neurons derived from fibroblasts collected from patients with type 1 and 2 GD with parkinsonism	NCGC607 (a non-inhibitory chaperone of GBA)	Lyso-Gb1 accumulated owing to GBA deficiency. NCGC607 significantly reduced lyso-Gb1 levels in cell lines of patients with GD
Ferraz et al., 2016 [5]	Fibroblasts obtained from patients with type 1 GD and controls	CBE or carmofur (an irreversible inhibitor of acid ceramidase)	Excessive lyso-Gb1 was demonstrated in cultured fibroblasts of a collodion patients with GD.Lyso-Gb1 levels were increased in the fibroblasts of patients with GD type 1.Lyso-Gb1 formation was induced by exposure to CBE and reduced by exposure to carmofur
**Animal**			
Xu et al., 2014 [6]	Newborn neural cells from a type 3 GD mouse model	CBE	CBE induced a 28-fold increase in lyso-Gb1 level
Westbroek et al., 2016 [7]	Immortalized cortical neurons from embryonic null allele *GBA*$^{-/-}$ mice and the control littermate (*GBA*$^{+/+}$)	None	Significant lyso-Gb1 storage was observed in *GBA*$^{-/-}$ vs. *GBA*$^{+/+}$ immortalized neurons (485.7 vs. 0.071 mg protein, respectively)
Taguchi et al., 2017 [8]	*GBA* mutant (N370S, L444P) and knockout mouse models crossed with an α-synuclein transgenic PD mouse	None	Lyso-Gb1 specifically accumulated in young GD/PD mouse brain

CBE: conduritol B epoxide; GBA: glucosylceramidase; GD: Gaucher disease; lyso-Gb1: glucosylsphingosine; PD Parkinson disease.

Table S5. In vivo studies reporting on lyso-Gb1 accumulation in GD models.

Author(s), Year [Reference]	Animal Model	Anatomical Compartment with Lyso-Gb1 Accumulation			
		Blood	Viscera[a]	Bone	Brain
**Pharmacologically Induced GD**					
Atsumi et al., 1993 [9]	Murine: wild type treated with cyclophellitol[b]		✓		✓
Rocha et al., 2015 [10]	Murine: wild type treated with CBE[c] or vehicle				✓[d]
Marshall et al., 2016 [11]	Murine: wild type treated with CBE				✓[e]
Vardi et al., 2016 [12]	Murine: wild type treated with CBE				✓
Hamler et al., 2017 [13]	Murine: wild type treated with CBE				✓
**Conditional Type 1 GD**					
Mistry et al., 2010 [14]	Murine: *GBA1* gene deletion in hematopoietic and mesenchymal lineages (knockout/LoxP/Mx1)		✓		
Sun et al., 2012 [15]	hG/4L/PS-NA		✓[f]		
Pavlova et al., 2013 [16]	Murine: $GBA^{tm1Karl/tm1Karl}$Tg(Mx1–Cre)1Cgn/0 vs. induced $GBA^{tm1Karl/tm1Karl}$ and $GBA^{tm1Karl/+}$ genotypes	✓	✓		
Mistry et al., 2014 [17]	Murine: double-mutant Mx1–Cre⁺:GD1:$GBA2^{-/-}$		✓		
Pavlova et al., 2015 [18]	Murine: $GBA^{tm1Karl/tm1Karl}$Tg(Mx1–Cre)1Cgn/0 vs. induced $GBA^{tm1Karl/tm1Karl}$ and $GBA^{tm1Karl/+}$ genotypes	✓[g]			
Dah et al.,l 2015 [19]	Murine: Mx1–Cre⁺ $GBA^{flox/flox}$		✓[h]	✓[h]	
Ferraz et al., 2016a [20]	Murine: $GBA^{tm1Karl/tm1Karl}$Tg(Mx1–Cre)1Cgn/0 with inducible knock down of *GBA* in the WBC lineage		✓		
**Neuronal GD**					
Cabrera-Salazar et al., 2010 & 2012 [21, 22]	Murine: K-14Cre⁺ $GBA^{lnl/lnl}$ vs. wild type				✓
Sun et al., 2011 [23]	Murine: 4L;C* (V394L/V394L + saposin $C^{-/-}$)		✓		✓
Farfel-Becker et al., 2013 [24]	Neuronopathic GD murine: $GBA^{flox/flox}$; nestin–Cre				✓
Vardi et al., 2016 [12]	Neuronopathic GD murine: $GBA^{flox/flox}$; nestin–Cre				✓
Bodennec et al., 2003 [25]	Type 2 murine: $GBA^{-/-}$ vs. $GBA^{+/+}$				✓
Enquist et al., 2007 [26]	Type 2 murine: K-14Cre+ $GBA^{lnl/lnl}$				✓
Karageorgos et al., 2016 [27]	Type 2 newborn lambs: homozygous C381Y vs. heterozygous C381Y and wild type		✓		✓

Smith et al., 2018 [28]	Type 2 murine: K-14Cre+ $GBA^{lnl/lnl}$ vs. wild type				✓
Sun et al., 2010 [14]	Type 3 (subacute) murine: transgenic 4L;C*[i]		✓		✓
Sun et al., 2013 [29]	Type 3 murine:D409V and null alleles (9V/null) (chronic) 9H (D409H);C* and 4L;C*[h] (subacute)	✓			✓
Barnes et al., 2014 [30]	Type 3 (chronic) murine: 9V/null allele with GCS transgene[j]		✓		✓
Dasgupta et al., 2015 [31]	Type 3 (subacute) murine: transgenic 4L;C*[i]				✓[k]
Dai et al., 2016 [32]	Type 3 (chronic) murine: D409V and null alleles (9V/null)				✓
Marshall et al., 2016 [11]	Type 3 (subacute) murine: transgenic 4L;C*[i]				✓[e]
Sardi et al., 2017 [33]	Type 3 (chronic) murine: D409V/D409 alleles (9V/9V)				✓[l]
Taguchi et al., 2017 [8]	GD/PD model: homozygous $GBA^{L444P/KO}$, $GBA^{N370S/KO}$, and $GBA^{KO/KO}$				✓
Zhang et al., 2017 [34]	Type 3 murine: transgenic 4L;C*[g] (subacute) vs. 9V/null allele (chronic)				✓[m]
Hamler et al., 2017 [13]	Type 3 murine: $GBA^{L444P/L444P}$, hL444P-tg8, hN370S-tg4, $PS^{+/+}$ NA 4L, and $PS^{-/-}$ NA 4L vs. wild type				✓

[a] Liver/spleen/lung.

[b] A GBA inhibitor

[c] A specific GBA inhibitor.

[d] Lyso-Gb1 levels were reduced in forebrain, midbrain, and cerebellum following removal of treatment over a period of 7 days.

[e] Reversed by ibiglustat, a specific inhibitor of GCS.

[f] Reversed by isofagomine, a potent GBA reversible competitive inhibitor and effective in vitro chaperone.

[g] Reversed by eliglustat, a potent and selective inhibitor of GCS.

[h] Reversed by lentiviral gene therapy, which drove expression of codon-optimized GBA complementary DNA.

[i] Harbors a homozygous V394L mutation in the GBA locus coupled with a knock-in mutation in the saposin C-encoding region.

[j] GCS transgene added to enhanced glycosphingolipid biosynthesis.

[k] Not reversed by isofagomine.

[l] Reversed by GZ667161, an inhibitor of GCS.

[m] Lyso-Gb1 level higher in transgenic 4L;C*g than in 9V/null allelic mice.

CBE: conduritol B epoxide; GBA: glucosylceramidase; GCS: glucosylceramide synthase; GD: Gaucher disease; lyso-Gb1: glucosylsphingosine; PD: Parkinson disease; WBC: white blood cell.

Table S6. Clinical studies reporting on lyso-Gb1 levels, as measured by liquid chromatography techniques, in GD during treatment with ERT and/or SRT.

Author(s), Year [Reference]	Study Design	Population Type	Treatment	Observation
Dekker et al., 2011 [35]	Observational, prospective, multicenter, longitudinal	Type 1 (N = 16)	ERT: imiglucerase (n = 14); SRT: miglustat (n = 2)	Marked reductions in plasma lyso-Gb1 levels in 9 of 14 patients receiving ERT. Effect of SRT alone was less prominent (n = 2)
Rolfs et al., 2013 [36]	Observational, prospective, single center, longitudinal	Mixed (N = 19) [a]	ERT: imiglucerase	Marked reductions from baseline in plasma lyso-Gb1 levels after start of ERT (from 200 ng/mL to < 50 ng/mL on average) and increases in plasma lyso-Gb1 levels when therapy discontinued
Mistry et al., 2014 [17]	Observational, prospective, cross-sectional, case control	Type 1 (n = 41), control (n = NR)	ERT: imiglucerase	Marked reductions from baseline in plasma lyso-Gb1 levels after start of ERT (from 181 ng/mL to 99 ng/mL) compared with controls (1.3 ng/mL)
Mirzaian et al., 2015 [37]	Observational, prospective, single center, longitudinal	Type 1 (N = 2)	ERT	Marked reductions from baseline in plasma and urine lyso-Gb1 levels after start of ERT
Fuller et al., 2015 [38]	Observational, prospective, single center, longitudinal	Mixed (N = 20)	ERT: velaglucerase (n = 11), taliglucerase (n = 6), imiglucerase (n = 1); untreated (n = 2)	Reductions from baseline in plasma lyso-Gb1 levels after start of ERT
Smid et al., 2016 [39]	Observational, retrospective, longitudinal	Type 1 treatment naïve and ERT experienced (N = 17)	ERT (n = 4); SRT: eliglustat (n = 6) or miglustat (n = 9)	After 2 years, median decrease of plasma lyso-Gb1 levels was 86%, 78%, and 48% for eliglustat-, ERT-, and miglustat-naïve patients, respectively
Murugesan et al., 2016 [40]	Observational, prospective, single center, longitudinal, case control	Type 1 *(N = 116)*	ERT: imiglucerase (n = 114); SRT: eliglustat (n = 14)	Marked reductions from baseline in plasma lyso-Gb1 levels after start of ERT (from 181 ng/mL to 89 ng/mL) compared with controls (1.5 ng/mL). By propensity scoring, SRT (n = 9) resulted in greater reduction of lyso-Gb1 than ERT (n = 47)
Mistry et al., 2017 [41]	Clinical trial, phase 3, randomized, multicenter, placebo-controlled, crossover	Type 1 treatment naïve (N = 40)	SRT: eliglustat	Plasma lyso-Gb1 levels decreased during 18 months' SRT, including in placebo crossover patients
Arkadir et al., 2018 [42]	Observational, retrospective, multicenter, longitudinal	Type 1 non-splenectomized N370S homozygotes (N = 25)	ERT: imiglucerase (n = 4), taliglucerase (n = 4), velaglucerase (n = 17)	Plasma lyso-Gb1 levels decreased during ERT over a median of 46 months' follow up

Lukina et al., 2019 [43]	Clinical trial, phase 2, multicenter	Type 1 treatment naïve (*N* = 26)	SRT: eliglustat for 8 years	Plasma lyso-Gb1 levels decreased by 92% after 8 years of SRT
Franco et al., 2017 [44]	Observational, prospective, multicenter, cross-sectional, case control	Type 1 and neuronopathic (*N* = 31)	ERT (*n* = 15); untreated (*n* = 16)	RBC membrane levels of lyso-Gb1 were lower in patients treated with ERT than in those not treated (0.69 vs. 0.34 pmol/mg of protein, respectively)
Elstein et al., 2017 [45]	Exploratory pooled analysis, phase 3 clinical trials	Type 1 treatment naïve (*n* = 22), ERT switch (*n* = 21)	ERT: velaglucerase	Plasma lyso-Gb1 levels were reduced in the treatment-naïve group (after 209 weeks) and switch groups (after 161 weeks) by 83% and 52%, respectively
Zhang et al., 2017 [34]	Observational, retrospective, multicenter, cross-sectional	Type 1 (*n* = 9), type 2 (*n* = 1), type 3 (*n* = 4)	ERT	Marked reductions from baseline in plasma lyso-Gb1 levels after start of ERT (from 86 nM to 31 nM), which were still elevated vs. healthy controls. Non-response in a patient with type 3 GD (L444P/L444P) on velaglucerase for 16 years since the age of 2 years
Murugesan et al., 2018 [46]	Observational, prospective, longitudinal	Type 1 (*N* = 41)	ERT: imiglucerase	Marked reductions in serum lyso-Gb1 levels after start of ERT (from 181 ng/mL to 99 ng/mL)
Tylki-Szymanska et al., 2018 [47]	Observational, prospective, single center, cross-sectional	Type 1 and neuronopathic (*N* = 64)	ERT (*n* = 56); untreated (*n* = 8)	Marked reductions in plasma lyso-Gb1 among ERT-treated vs. untreated patients (114 vs. 655 ng/mL, respectively)
Narita et al., 2016 [48]	Clinical trial, multicenter, open-label, pilot	Neuronopathic (*n* = 5), healthy controls (*n* = 37)	ERT plus ambroxol[b]	CSF lyso-Gb1 levels fell by 26% vs. baseline. CSF lyso-Gb1 levels were below the lower limit of quantification (10.0 pg/mL) in all control subjects

[a] All subjects were non-Jewish and caucasian.

[b] Enhances endogenous GBA activity in the murine central nervous system [49].

CSF: cerebrospinal fluid; ERT: enzyme replacement therapy; GBA: glucosylceramidase; GD: Gaucher disease; lyso-Gb1: glucosylsphingosine; NR: not reported; RBC: red blood cell; SRT: substrate reduction therapy.

References.

1. Sasagasako, N.; Kobayashi, T.; Yamaguchi, Y.; Shinnoh, N.; Goto, I. Glucosylceramide and glucosylsphingosine metabolism in cultured fibroblasts deficient in acid beta-glucosidase activity. *J. Biochem.* **1994**, *115*, 113–119.
2. Aflaki, E.; Stubblefield, B.K.; Maniwang, E.; Lopez, G.; Moaven, N.; Goldin, E.; Marugan, J.; Patnaik, S.; Dutra, A.; Southall, N.; et al. Macrophage models of Gaucher disease for evaluating disease pathogenesis and candidate drugs. *Sci. Transl. Med.* **2014**, *6*, 240ra73.
3. Sun, Y.; Florer, J.; Mayhew, C.N.; Jia, Z.; Zhao, Z.; Xu, K.; Ran, H.; Liou, B.; Zhang, W.; Setchell, K.D.; et al. Properties of neurons derived from induced pluripotent stem cells of Gaucher disease type 2 patient fibroblasts: Potential role in neuropathology. *PLoS ONE* **2015**, *10*, e0118771.
4. Aflaki, E.; Borger, D.K.; Moaven, N.; Stubblefield, B.K.; Rogers, S.A.; Patnaik, S.; Schoenen, F.J.; Westbroek, W.; Zheng, W.; Sullivan, P.; et al. A new glucocerebrosidase chaperone reduces α-synuclein and glycolipid levels in iPSC-derived dopaminergic neurons from patients with Gaucher disease and Parkinsonism. *J. Neurosci.* **2016**, *36*, 7441–7452.
5. Ferraz, M.J.; Marques, A.R.; Appelman, M.D.; Verhoek, M.; Strijland, A.; Mirzaian, M.; Scheij, S.; Ouairy, C.M.; Lahav, D.; Wisse, P.; et al. Lysosomal glycosphingolipid catabolism by acid ceramidase: Formation of glycosphingoid bases during deficiency of glycosidases. *Febs Lett.* **2016**, *590*, 716–725.
6. Xu, Y.H.; Xu, K.; Sun, Y.; Liou, B.; Quinn, B.; Li, R.H.; Xue, L.; Zhang, W.; Setchell, K.D.; Witte, D.; et al. Multiple pathogenic proteins implicated in neuronopathic Gaucher disease mice. *Hum. Mol. Genet.* **2014**, *23*, 3943–3957.
7. Westbroek, W.; Nguyen, M.; Siebert, M.; Lindstrom, T.; Burnett, R.A.; Aflaki, E.; Jung, O.; Tamargo, R.; Rodriguez-Gil, J.L.; Acosta, W.; et al. A new glucocerebrosidase-deficient neuronal cell model provides a tool to probe pathophysiology and therapeutics for Gaucher disease. *Dis. Model. Mech.* **2016**, *9*, 769–778.
8. Taguchi, Y.V.; Liu, J.; Ruan, J.; Pacheco, J.; Zhang, X.; Abbasi, J.; Keutzer, J.; Mistry, P.K.; Chandra, S.S. Glucosylsphingosine promotes α-synuclein pathology in mutant GBA-associated Parkinson's disease. *J. Neurosci.* **2017**, *37*, 9617–9631.
9. Atsumi, S.; Nosaka, C.; Iinuma, H.; Umezawa, K. Accumulation of tissue glucosylsphingosine in Gaucher-like mouse induced by the glucosylceramidase inhibitor cyclophellitol. *Arch. Biochem. Biophys.* **1993**, *304*, 302–304.
10. Rocha, E.M.; Smith, G.A.; Park, E.; Cao, H.; Graham, A.R.; Brown, E.; McLean, J.R.; Hayes, M.A.; Beagan, J.; Izen, S.C.; et al. Sustained systemic glucocerebrosidase inhibition induces brain α-synuclein aggregation, microglia and complement C1q activation in mice. *Antioxid. Redox Signal.* **2015**, *23*, 550–564.
11. Marshall, J.; Sun, Y.; Bangari, D.S.; Budman, E.; Park, H.; Nietupski, J.B.; Allaire, A.; Cromwell, M.A.; Wang, B.; Grabowski, G.A.; et al. CNS-accessible inhibitor of glucosylceramide synthase for substrate reduction therapy of neuronopathic Gaucher disease. *Mol.* . **2016**, *24*, 1019–1029.
12. Vardi, A.; Zigdon, H.; Meshcheriakova, A.; Klein, A.D.; Yaacobi, C.; Eilam, R.; Kenwood, B.M.; Rahim, A.A.; Massaro, G.; Merrill, A.H., Jr.; et al. Delineating pathological pathways in a chemically induced mouse model of Gaucher disease. *J. Pathol.* **2016**, *239*, 496–509.
13. Hamler, R.; Brignol, N.; Clark, S.W.; Morrison, S.; Dungan, L.B.; Chang, H.H.; Khanna, R.; Frascella, M.; Valenzano, K.J.; Benjamin, E.R.; et al. Glucosylceramide and glucosylsphingosine quantitation by liquid chromatography-tandem mass spectrometry to enable in vivo preclinical studies of neuronopathic Gaucher disease. *Anal. Chem.* **2017**, *89*, 8288–8295.
14. Mistry, P.K.; Liu, J.; Yang, M.; Nottoli, T.; McGrath, J.; Jain, D.; Zhang, K.; Keutzer, J.; Chuang, W.L.; Mehal, W.Z.; et al. Glucocerebrosidase gene-deficient mouse recapitulates Gaucher disease displaying cellular and molecular dysregulation beyond the macrophage. *Proc. Natl. Acad. Sci. U. S. A.* **2010**, *107*, 19473–19478.
15. Sun, Y.; Liou, B.; Xu, Y.H.; Quinn, B.; Zhang, W.; Hamler, R.; Setchell, K.D.; Grabowski, G.A. Ex vivo and in vivo effects of isofagomine on acid β-glucosidase variants and substrate levels in Gaucher disease. *J. Biol. Chem.* **2012**, *287*, 4275–4287.
16. Pavlova, E.V.; Wang, S.Z.; Archer, J.; Dekker, N.; Aerts, J.M.; Karlsson, S.; Cox, T.M. B cell lymphoma and myeloma in murine Gaucher's disease. *J. Pathol.* **2013**, *231*, 88–97.

17. Mistry, P.K.; Liu, J.; Sun, L.; Chuang, W.L.; Yuen, T.; Yang, R.; Lu, P.; Zhang, K.; Li, J.; Keutzer, J.; et al. Glucocerebrosidase 2 gene deletion rescues type 1 Gaucher disease. *Proc. Natl. Acad. Sci. U. S. A.* **2014**, *111*, 4934–4939.
18. Pavlova, E.V.; Archer, J.; Wang, S.; Dekker, N.; Aerts, J.M.; Karlsson, S.; Cox, T.M. Inhibition of UDP-glucosylceramide synthase in mice prevents Gaucher disease-associated B-cell malignancy. *J. Pathol.* **2015**, *235*, 113–124.
19. Dahl, M.; Doyle, A.; Olsson, K.; Månsson, J.E.; Marques, A.R.A.; Mirzaian, M.; Aerts, J.M.; Ehinger, M.; Rothe, M.; Modlich, U.; et al. Lentiviral gene therapy using cellular promoters cures type 1 Gaucher disease in mice. *Mol.* . **2015**, *23*, 835–844.
20. Ferraz, M.J.; Marques, A.R.; Gaspar, P.; Mirzaian, M.; van Roomen, C.; Ottenhoff, R.; Alfonso, P.; Irún, P.; Giraldo, P.; Wisse, P.; et al. Lyso-glycosphingolipid abnormalities in different murine models of lysosomal storage disorders. *Mol. Genet. Metab.* **2016**, *117*, 186–193.
21. Cabrera-Salazar, M.A.; Bercury, S.D.; Ziegler, R.J.; Marshall, J.; Hodges, B.L.; Chuang, W.L.; Pacheco, J.; Li, L.; Cheng, S.H.; Scheule, R.K. Intracerebroventricular delivery of glucocerebrosidase reduces substrates and increases lifespan in a mouse model of neuronopathic Gaucher disease. *Exp. Neurol.* **2010**, *225*, 436–444.
22. Cabrera-Salazar, M.A.; Deriso, M.; Bercury, S.D.; Li, L.; Lydon, J.T.; Weber, W.; Pande, N.; Cromwell, M.A.; Copeland, D.; Leonard, J.; et al. Systemic delivery of a glucosylceramide synthase inhibitor reduces CNS substrates and increases lifespan in a mouse model of type 2 Gaucher disease. *PLoS ONE* **2012**, *7*, e43310.
23. Sun, Y.; Ran, H.; Liou, B.; Quinn, B.; Zamzow, M.; Zhang, W.; Bielawski, J.; Kitatani, K.; Setchell, K.D.; Hannun, Y.A.; et al. Isofagomine in vivo effects in a neuronopathic Gaucher disease mouse. *PLoS ONE* **2011**, *6*, e19037.
24. Farfel-Becker, T.; Vitner, E.B.; Kelly, S.L.; Bame, J.R.; Duan, J.; Shinder, V.; Merrill, A.H., Jr.; Dobrenis, K.; Futerman, A.H. Neuronal accumulation of glucosylceramide in a mouse model of neuronopathic Gaucher disease leads to neurodegeneration. *Hum. Mol. Genet.* **2014**, *23*, 843–854.
25. Bodennec, J.; Trajkovic-Bodennec, S.; Futerman, A.H. Simultaneous quantification of lyso-neutral glycosphingolipids and neutral glycosphingolipids by N-acetylation with [3H]acetic anhydride. *J. Lipid Res.* **2003**, *44*, 1413–1419.
26. Enquist, I.B.; Lo Bianco, C.; Ooka, A.; Nilsson, E.; Månsson, J.E.; Ehinger, M.; Richter, J.; Brady, R.O.; Kirik, D.; Karlsson, S. Murine models of acute neuronopathic Gaucher disease. *Proc. Natl. Acad. Sci. U. S. A.* **2007**, *104*, 17483–17488.
27. Karageorgos, L.; Hein, L.; Rozaklis, T.; Adams, M.; Duplock, S.; Snel, M.; Hemsley, K.; Kuchel, T.; Smith, N.; Hopwood, J.J. Glycosphingolipid analysis in a naturally occurring ovine model of acute neuronopathic Gaucher disease. *Neurobiol. Dis.* **2016**, *91*, 143–154.
28. Smith, N.J.; Fuller, M.; Saville, J.T.; Cox, T.M. Reduced cerebral vascularization in experimental neuronopathic Gaucher disease. *J. Pathol.* **2018**, *244*, 120–128.
29. Sun, Y.; Zhang, W. Glycosphingolipid aspects of Gaucher disease lipidomics. In *Advances in Gaucher Disease: Basic and Clinical Perspectives*; Future Medicine: London, UK, 2013; pp. 94–106.
30. Barnes, S.; Xu, Y.H.; Zhang, W.; Liou, B.; Setchell, K.D.; Bao, L.; Grabowski, G.A.; Sun, Y. Ubiquitous transgene expression of the glucosylceramide-synthesizing enzyme accelerates glucosylceramide accumulation and storage cells in a Gaucher disease mouse model. *PLoS ONE* **2014**, *9*, e116023.
31. Dasgupta, N.; Xu, Y.H.; Li, R.; Peng, Y.; Pandey, M.K.; Tinch, S.L.; Liou, B.; Inskeep, V.; Zhang, W.; Setchell, K.D.; et al. Neuronopathic Gaucher disease: Dysregulated mRNAs and miRNAs in brain pathogenesis and effects of pharmacologic chaperone treatment in a mouse model. *Hum. Mol. Genet.* **2015**, *24*, 7031–7048.
32. Dai, M.; Liou, B.; Swope, B.; Wang, X.; Zhang, W.; Inskeep, V.; Grabowski, G.A.; Sun, Y.; Pan, D. Progression of behavioral and CNS deficits in a viable murine model of chronic neuronopathic Gaucher disease. *PLoS ONE* **2016**, *11*, e0162367.
33. Sardi, S.P.; Viel, C.; Clarke, J.; Treleaven, C.M.; Richards, A.M.; Park, H.; Olszewski, M.A.; Dodge, J.C.; Marshall, J.; Makino, E.; et al. Glucosylceramide synthase inhibition alleviates aberrations in synucleinopathy models. *Proc. Natl. Acad. Sci. U. S. A.* **2017**, *114*, 2699–2704.
34. Zhang, W.; Oehrle, M.; Prada, C.E.; Schwartz, I.V.D.; Chutipongtanate, S.; Wattanasirichaigoon, D.; Inskeep, V.; Dai, M.; Pan, D.; Sun, Y.; et al. A convenient approach to facilitate monitoring Gaucher disease progression and therapeutic response. *Analyst* **2017**, *142*, 3380–3387.

35. Dekker, N.; van Dussen, L.; Hollak, C.E.; Overkleeft, H.; Scheij, S.; Ghauharali, K.; van Breemen, M.J.; Ferraz, M.J.; Groener, J.E.; Maas, M.; et al. Elevated plasma glucosylsphingosine in Gaucher disease: Relation to phenotype, storage cell markers, and therapeutic response. *Blood* **2011**, *118*, e118–e127.
36. Rolfs, A.; Giese, A.K.; Grittner, U.; Mascher, D.; Elstein, D.; Zimran, A.; Böttcher, T.; Lukas, J.; Hübner, R.; Gölnitz, U.; et al. Glucosylsphingosine is a highly sensitive and specific biomarker for primary diagnostic and follow-up monitoring in Gaucher disease in a non-Jewish, Caucasian cohort of Gaucher disease patients. *PLoS ONE* **2013**, *8*, e79732.
37. Mirzaian, M.; Wisse, P.; Ferraz, M.J.; Gold, H.; Donker-Koopman, W.E.; Verhoek, M.; Overkleeft, H.S.; Boot, R.G.; Kramer, G.; Dekker, N.; et al. Mass spectrometric quantification of glucosylsphingosine in plasma and urine of type 1 Gaucher patients using an isotope standard. *Blood Cells Mol. Dis.* **2015**, *54*, 307–314.
38. Fuller, M.; Szer, J.; Stark, S.; Fletcher, J.M. Rapid, single-phase extraction of glucosylsphingosine from plasma: A universal screening and monitoring tool. *Clin. Chim. Acta* **2015**, *450*, 6–10.
39. Smid, B.E.; Ferraz, M.J.; Verhoek, M.; Mirzaian, M.; Wisse, P.; Overkleeft, H.S.; Hollak, C.E.; Aerts, J.M. Biochemical response to substrate reduction therapy versus enzyme replacement therapy in Gaucher disease type 1 patients. *Orphanet J. Rare Dis.* **2016**, *11*, 28.
40. Murugesan, V.; Chuang, W.L.; Liu, J.; Lischuk, A.; Kacena, K.; Lin, H.; Pastores, G.M.; Yang, R.; Keutzer, J.; Zhang, K.; et al. Glucosylsphingosine is a key biomarker of Gaucher disease. *Am. J. Hematol.* **2016**, *91*, 1082–1089.
41. Mistry, P.K.; Lukina, E.; Ben Turkia, H.; Shankar, S.P.; Baris, H.; Ghosn, M.; Mehta, A.; Packman, S.; Pastores, G.; Petakov, M.; et al. Outcomes after 18 months of eliglustat therapy in treatment-naïve adults with Gaucher disease type 1: The phase 3 ENGAGE trial. *Am. J. Hematol.* **2017**, *92*, 1170–1176.
42. Arkadir, D.; Dinur, T.; Revel-Vilk, S.; Becker Cohen, M.; Cozma, C.; Hovakimyan, M.; Eichler, S.; Rolfs, A.; Zimran, A. Glucosylsphingosine is a reliable response biomarker in Gaucher disease. *Am. J. Hematol.* **2018**, *93*, E140–E142.
43. Lukina, E.; Watman, N.; Dragosky, M.; Lau, H.; Avila Arreguin, E.; Rosenbaum, H.; Zimran, A.; Foster, M.C.; Gaemers, S.J.M.; Peterschmitt, M.J. Outcomes after 8 years of eliglustat therapy for Gaucher disease type 1: Final results from the Phase 2 trial. *Am. J. Hematol.* **2019**, *94*, 29–38.
44. Franco, M.; Reihani, N.; Marin, M.; De Person, M.; Billette de Villemeur, T.; Rose, C.; Colin, Y.; Moussa, F.; Belmatoug, N.; Le Van Kim, C. Effect of velaglucerase alfa enzyme replacement therapy on red blood cell properties in Gaucher disease. *Am. J. Hematol.* **2017**, *92*, E561–E563.
45. Elstein, D.; Mellgard, B.; Dinh, Q.; Lan, L.; Qiu, Y.; Cozma, C.; Eichler, S.; Bottcher, T.; Zimran, A. Reductions in glucosylsphingosine (lyso-Gb1) in treatment-naïve and previously treated patients receiving velaglucerase alfa for type 1 Gaucher disease: Data from phase 3 clinical trials. *Mol. Genet. Metab.* **2017**, *122*, 113–120.
46. Murugesan, V.; Liu, J.; Yang, R.; Lin, H.; Lischuk, A.; Pastores, G.; Zhang, X.; Chuang, W.L.; Mistry, P.K. Validating glycoprotein non-metastatic melanoma B (gpNMB, osteoactivin), a new biomarker of Gaucher disease. *Blood Cells Mol. Dis.* **2018**, *68*, 47–53.
47. Tylki-Szymańska, A.; Szymańska-Rożek, P.; Hasiński, P.; Ługowska, A. Plasma chitotriosidase activity versus plasma glucosylsphingosine in wide spectrum of Gaucher disease phenotypes-a statistical insight. *Mol. Genet. Metab.* **2018**, *123*, 495–500.
48. Narita, A.; Shirai, K.; Itamura, S.; Matsuda, A.; Ishihara, A.; Matsushita, K.; Fukuda, C.; Kubota, N.; Takayama, R.; Shigematsu, H.; et al. Ambroxol chaperone therapy for neuronopathic Gaucher disease: A pilot study. *Ann. Clin. Transl. Neurol.* **2016**, *3*, 200–215.
49. Luan, Z.; Li, L.; Higaki, K.; Nanba, E.; Suzuki, Y.; Ohno, K. The chaperone activity and toxicity of ambroxol on Gaucher cells and normal mice. *Brain Dev.* **2013**, *35*, 317–322.

.
